# Antibody-based regimens targeting PD-1/PD-L1 and VEGF/VEGFR in advanced or metastatic NSCLC: a meta-analysis of RCTs

**DOI:** 10.3389/fimmu.2026.1847913

**Published:** 2026-06-09

**Authors:** Wenjie Mao, Zeqi Tang, Yu Chen, Yixia Chen, Shiyan Zhang, Xiaoling Feng, Shengying Zhang

**Affiliations:** 1 Department of Pulmonary and Critical Care Medicine, Ningbo Hospital of Integrated Traditional Chinese and Western Medicine, Ningbo, Zhejiang, China; 2 The Fifth School of Clinical Medical, Zhejiang Chinese Medical University, Hangzhou, Zhejiang, China; 3 The Graduate School, Zhejiang Chinese Medical University, Hangzhou, Zhejiang, China; 4 The Second Clinical Medical College, Zhejiang Chinese Medical University, Hangzhou, Zhejiang, China

**Keywords:** anti-angiogenic monoclonal antibodies, meta-analysis, NSCLC, PD-1/PD-L1 inhibitors, prognosis

## Abstract

**Background:**

Antibody-based regimens targeting programmed cell death protein-1 (PD-1)/programmed death-ligand 1 (PD-L1) and vascular endothelial growth factor (VEGF)/vascular endothelial growth factor receptor (VEGFR) include conventional PD-1/PD-L1 inhibitors combined with anti-angiogenic monoclonal antibodies and PD-1/VEGF or PD-L1/VEGF bispecific antibodies, with or without chemotherapy. However, the clinical efficacy of these regimens in advanced or metastatic non-small cell lung cancer (NSCLC) remains unclear. This meta-analysis systematically evaluated their efficacy.

**Materials and methods:**

PubMed, EMBASE, Web of Science, ClinicalTrials.gov, and the Cochrane Library were searched to identify relevant randomized controlled trials (RCTs) published up to February 2026. The main outcomes analyzed were progression-free survival (PFS) and overall survival (OS), with the hazard ratio (HR) and 95% confidence interval (95%CI) used for statistical analysis. The study protocol was registered on PROSPERO (CRD420251126421).

**Results:**

Eleven RCTs were included in the analysis, with 4,426 participants. Antibody-based regimens targeting PD-1/PD-L1 and VEGF/VEGFR, with or without chemotherapy, were associated with better PFS (HR = 0.65, 95%CI: 0.57-0.75, p<0.001) and OS (HR = 0.79, 95%CI: 0.71-0.87, p<0.001) compared with the control regimen. For PFS, favorable subgroup estimates were observed in men (HR = 0.61), squamous histology (HR = 0.57), patients with liver metastases (HR = 0.49), those with higher PD-L1 expression (tumor proportion score (TPS)≥50%: HR = 0.65), those with epidermal growth factor receptor (EGFR) mutation (HR = 0.60). For OS, favorable subgroup estimates were observed in patients with Eastern Cooperative Oncology Group (ECOG) performance status (PS)=1 (HR = 0.80), men (HR = 0.80), smokers (HR = 0.82), those with liver metastases (HR = 0.57), those with PD-L1 TPS 1%-49% (HR = 0.67), and EGFR-mutant patients (HR = 0.80).

**Conclusion:**

Antibody-based regimens targeting PD-1/PD-L1 and VEGF/VEGFR improve prognosis in advanced or metastatic NSCLC, with potential variation in benefit across clinicopathological characteristics.

## 1 Introduction

Lung cancer remains one of the most common malignancies worldwide and is associated with a substantial disease burden, accounting for 12.4% of all new cancer cases and 18.7% of cancer-related deaths globally in 2022 (1). Non-small cell lung cancer (NSCLC) represents approximately 85% of all lung cancers. Owing to the paucity of specific symptoms at early stages, more than half of patients with NSCLC (around 55%) present with distant metastases at initial diagnosis, whereas only approximately 20% have localized disease, which significantly contributes to the poor prognosis of these patients (2, 3).

Treatment strategies for advanced or metastatic NSCLC are largely guided by the presence of actionable oncogenic alterations. For patients without targetable driver mutations, immune checkpoint inhibitors (ICIs), particularly programmed cell death protein-1 (PD-1)/programmed death-ligand 1 (PD-L1) inhibitors, have become an important component of first-line therapy and have been shown to improve survival (4, 5). However, the efficacy of PD-1/PD-L1 inhibitor monotherapy remains limited by modest response rates (6). In patients with NSCLC unselected by PD-L1 expression, the objective response rate is approximately 20% (7). Therefore, combination strategies incorporating PD-1/PD-L1 inhibitors have been widely explored.

In contrast, patients with targetable driver mutations usually receive targeted therapy as the preferred first-line treatment. Epidermal growth factor receptor (EGFR) mutations are the most common actionable driver alterations in NSCLC, with exon 19 deletion and L858R accounting for most sensitizing EGFR mutations (8). Although EGFR-tyrosine kinase inhibitors (TKIs) provide substantial initial benefit, most patients eventually develop acquired resistance. After failure of first- or second-generation EGFR-TKIs, EGFR T790M is one of the most common resistance mechanisms, occurring in approximately 40%-50% of cases (9). After EGFR-TKI failure, PD-1/PD-L1 inhibitor monotherapy has shown limited efficacy (10), and its benefit may vary according to molecular features, including EGFR mutation subtype, such as exon 19 deletion versus L858R (11), and T790M status (11, 12). These challenges highlight the need for more effective PD-1/PD-L1 inhibitor-based combination strategies after EGFR-TKI resistance.

Among potential partners for PD-1/PD-L1 blockade, inhibition of the vascular endothelial growth factor (VEGF)/vascular endothelial growth factor receptor (VEGFR) pathway has a strong biological rationale in advanced or metastatic NSCLC. Angiogenesis and tumor immunity are closely interconnected: VEGF/VEGFR signaling promotes abnormal tumor angiogenesis, impairs vascular normalization, and contributes to tumor growth (13). Beyond its pro-angiogenic role, VEGF also contributes to immunosuppression by inhibiting dendritic-cell (DC) maturation, impairing effector T-cell function, and limiting immune-cell infiltration into the tumor microenvironment (14). Therefore, inhibition of the VEGF/VEGFR pathway may improve tumor vascular function, alleviate VEGF-mediated immunosuppression, and enhance the antitumor activity of PD-1/PD-L1 blockade. This mechanism is relevant not only to advanced or metastatic NSCLC broadly, but also to EGFR-mutant NSCLC after resistance to EGFR-TKIs, where PD-1/PD-L1 inhibitor monotherapy has limited efficacy (15). Clinically, this strategy can be delivered through antibody-based regimens targeting PD-1/PD-L1 and VEGF/VEGFR. These include conventional combinations of PD-1/PD-L1 inhibitors with anti-angiogenic monoclonal antibodies, such as bevacizumab or ramucirumab, and bispecific antibodies targeting PD-1/PD-L1 and VEGF. Among the latter, PD-1/VEGF bispecific antibodies, such as ivonescimab, have been more extensively evaluated in studies (16). Early clinical studies have suggested that these antibody-based regimens may improve outcomes in advanced or metastatic NSCLC (17–19).

A meta-analysis by Duan et al. reported the efficacy of PD-1/PD-L1 inhibitors combined with anti-angiogenic agents in advanced NSCLC (20). However, most included studies were retrospective, and only five randomized controlled trials (RCTs) were available, which may limit the strength of the evidence. More importantly, the anti-angiogenic agents pooled in that analysis included both anti-angiogenic monoclonal antibodies and multi-target anti-angiogenic TKIs. Given their distinct mechanisms of action, such pooling may obscure the contribution of anti-angiogenic monoclonal antibodies. In addition, emerging PD-1/VEGF or PD-L1/VEGF bispecific antibodies were not specifically addressed in that analysis.

To address these evidence gaps, we conducted an updated meta-analysis of RCTs evaluating antibody-based regimens targeting PD-1/PD-L1 and VEGF/VEGFR, including conventional PD-1/PD-L1 inhibitor plus anti-angiogenic monoclonal antibody combinations and PD-1/VEGF or PD-L1/VEGF bispecific antibodies, with or without chemotherapy, in advanced or metastatic NSCLC. This meta-analysis aimed to assess the efficacy of these regimens and to provide more definitive evidence to inform clinical decision-making and future research.

## 2 Materials and methods

### 2.1 Search strategy

RCTs concerning the effectiveness of conventional PD-1/PD-L1 inhibitors combined with anti-angiogenic monoclonal antibodies or PD-1/VEGF or PD-L1/VEGF bispecific antibodies, with or without chemotherapy for patients with advanced or metastatic NSCLC were searched through five online databases (EMBASE, PubMed, Web of Science, the Cochrane library and ClinicalTrials.gov) from their inception to February 2026. A search strategy was formulated in accordance with the PICOS principles, incorporating both medical subject headings (MeSH) and free-text terms, such as ((Immune Checkpoint Inhibitors OR Programmed Cell Death Protein 1 inhibitors OR Programmed Death-Ligand 1 inhibitors OR Sintilimab OR Atezolizumab OR Durvalumab OR Nivolumab OR Pembrolizumab OR Toripalimab OR Penpulimab OR Serplulimab OR Adebrelimab OR Pucotenlimab) AND (Angiogenesis Inhibitors OR Anti-Angiogenetic Agents OR Bevacizumab OR Ramucirumab) OR (PD-1/VEGF bispecific antibody OR PD-L1/VEGF bispecific antibody OR Ivonescimab)) AND (Carcinoma, Non-Small-cell lung OR Non-Small Cell Lung Cancer OR Lung Carcinoma, Non-Small-Cell). And the detailed database-specific search strategies are provided in **Supplementary Search Strategy**. Meanwhile, references from published studies were retrieved to avoid potential omissions. This meta-analysis adhered to the guidelines and checklists of Preferred Reporting Items for Systematic and Meta-Analyses (PRISMA), with the study protocol prospectively registered in the International Prospective Register of Systematic Reviews (PROSPERO, identifier: CRD420251126421).

### 2.2 Eligibility criteria

Inclusion criteria: (1) Population: patients>18 years old, with locally advanced, metastatic or recurrent NSCLC. (2) Intervention: PD-1/PD-L1 inhibitors combined with anti-angiogenic monoclonal antibodies, or PD-1/VEGF or PD-L1/VEGF bispecific antibodies, with or without chemotherapy. (3) Comparison: any regimen without concomitant PD-1/PD-L1 inhibitors and anti-angiogenic monoclonal antibodies or PD-1/VEGF or PD-L1/VEGF bispecific antibodies, including PD-1/PD-L1 inhibitors (with or without chemotherapy), anti-angiogenic monoclonal antibodies (with or without chemotherapy) or chemotherapy as a standalone treatment. (4) Outcome: each included study assessed at least one of the following endpoints: progression-free survival (PFS) or overall survival (OS). (5) Study design: the study belongs to Phase II or Phase III RCT.

Exclusion criteria: (1) Sample size in any treatment arm<30, (2) non-English language publications, (3) for studies with multiple publications, only the most recent and comprehensive dataset was included, (4) inaccessible full text and unavailable data, (5) only the median survival times of OS and PFS were reported.

### 2.3 Data extraction

Following study screening per inclusion criteria, retrieved study was imported into EndNote 21 and duplicates were removed. Then, titles and abstracts of remaining records underwent independent screening by two authors to pinpoint articles warranting full-text review. Subsequently, two authors assessed the full text and extracted data independently. Disagreements were settled by consensus or third-party arbitration.

Data extraction from included articles utilized pre-set tables. The extracted data encompassed study characteristics (authors, publication year, trial name, clinical trial number, country, median follow-up time, recruiting time), patient’s baseline characteristics (sample size, EGFR mutation status, histology), intervention information (drug regimens, treatment line) and primary outcomes.

### 2.4 Risk of bias and quality assessments

The included RCTs were evaluated for methodological quality and potential bias with the Cochrane Risk of Bias tool. This evaluation covered the following key areas: random sequence generation (selection bias), allocation concealment (selection bias), blinding of participants and personnel (detection bias), blinding of outcome assessment (detection bias), handling of incomplete outcome data (attrition bias), selective reporting (reporting bias) and other bias. Two reviewers carried out the assessments independently, and any inconsistencies were settled through discussion or by a third researcher.

### 2.5 Statistical analysis

Statistical analysis was performed with Stata 12.0. Hazard ratios (HR) with 95% confidence intervals (95%CI) were the effect measures for OS and PFS. An HR<1 was interpreted as supporting the experimental group, whereas an HR>1 supported the control group. Heterogeneity was quantified using the I² statistic, with higher values indicating greater heterogeneity. We categorized heterogeneity as low (I²<25%), moderate (25%-50%), or high (I²>50%). In this meta-analysis, an I² value>50% was considered to indicate high heterogeneity. A random-effects model was employed for the pooled analysis to provide a more conservative estimate of the pooled effect and enhance the generalizability of the results, due to potential heterogeneity among the included studies in terms of populations, interventions, and outcome measurements. Prespecified subgroups encompassed age, sex, Eastern Cooperative Oncology Group (ECOG) performance status (PS), tobacco history, metastasis status, PD-L1 expression, EGFR mutation profile. Additionally, Begg’s test was used to analyze publication bias, and a leave-one-out sensitivity analysis was performed to test the robustness of the outcomes. All statistical tests were two-sided tests and were statistically significant when p<0.05.

## 3 Results

### 3.1 Study search results

**Figure 1:**
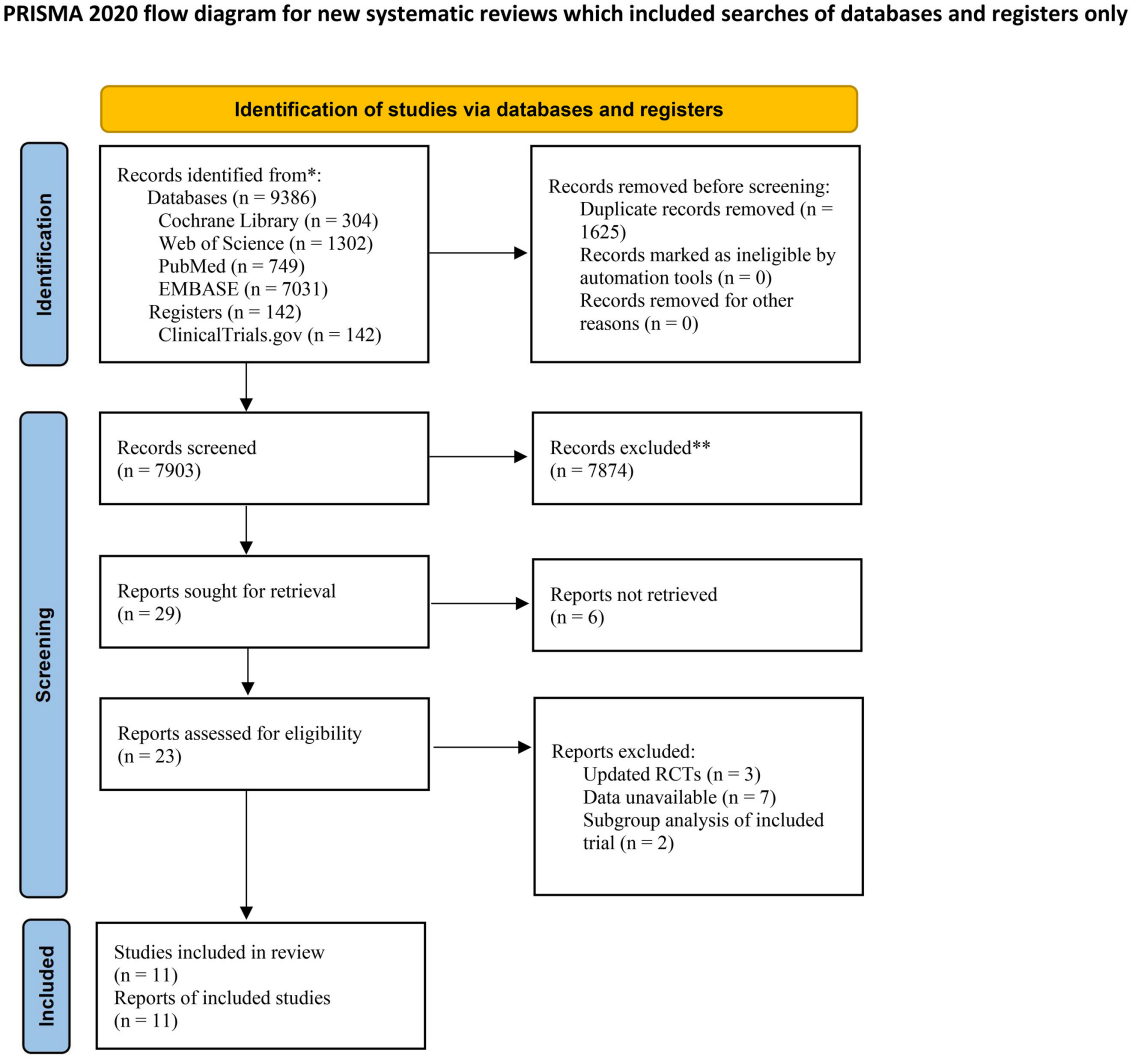
Flow chart representing details of the study selection. *Consider, if feasible to do so, reporting the number of records identified from each database or register searched (rather than the total number across all databases/registers). **If automation tools were used, indicate how many records were excluded by a human and how many were excluded by automation tools.

Without consulting any other sources, a comprehensive search produced 9,528 records from five databases: the Cochrane Library, Web of Science, PubMed, EMBASE, and ClinicalTrials.gov. There were 7,903 records left after deduplication. By reviewing the titles and abstracts of these studies, 7,874 records were excluded, and 29 reports were sought for retrieval. Among these, 6 reports were not retrieved because full texts were unavailable. The remaining 23 reports underwent full-text assessment, after which 12 were excluded: updated RCTs (n=3), unavailable data (n=7), and subgroup analyses of already included studies (n=2). Ultimately, 11 RCTs were included in the meta-analysis (17–19, 21–28), as shown in **Figure 1**.

### 3.2 Characteristics of studies and participants

Across these eleven RCTs, a total of 4,426 participants were involved, with 2,250 individuals in the intervention group and 2,176 in the control group. The studies were published between 2019 and 2026. Patients in the intervention group received antibody-based regimens targeting PD-1/PD-L1 and VEGF/VEGFR, with or without chemotherapy, including conventional PD-1/PD-L1 inhibitors plus anti-angiogenic monoclonal antibodies and PD-1/VEGF bispecific antibodies. PD-1/PD-L1 inhibitors included sintilimab, atezolizumab, pembrolizumab, nivolumab and serplulimab; anti-angiogenic monoclonal antibodies included bevacizumab, ramucirumab, IBI305 (bevacizumab biosimilar) and HLX04 (bevacizumab biosimilar). In three studies, patients received ivonescimab, a PD-1/VEGF bispecific antibody. The control group received chemotherapy, PD-1/PD-L1 inhibitors, or anti-angiogenic monoclonal antibodies, either alone or in combination with chemotherapy. The treatment settings included in the studies were as follows: four studies focused on first-line therapy, four studies targeted second-line or later therapy. The median follow-up period varied from 7.9 to 26.1 months across the studies. Six studies included patients with EGFR-mutant NSCLC, of which two exclusively enrolled EGFR-mutant patients. Eight studies exclusively enrolled patients with histologically confirmed non-squamous NSCLC. Details about the study characteristics and participant characteristics can be found in **Table 1** and **Supplementary Table 1**.

**Table 1:** Basic characteristics of all the randomized controlled trials included in this meta-analysis.

Author	Year	Trial name	Treatment line	Intervene			Control			Primary outcomes
				Sample size	Regimen	EGFR mutation status	Sample size	Regimen	EGFR mutation status	
Lu, S.	2023	ORIENT-31	≥2	158	Sintilimab + IBI305 + Chemotherapy	All mut	160	Placebo1 + Placebo2 + Chemotherapy	All mut	PFS
Reck, M.	2019	IMpower150	1/2	400	Atezolizumab + Bevacizumab + Chemotherapy	9% mut	400	Bevacizumab + Chemotherapy	11% mut	OS, PFS
Shiraishi, Y.	2024	APPLE	1/2	205	Atezolizumab + Bevacizumab + Chemotherapy	26% mut	206	Atezolizumab + Chemotherapy	29% mut	PFS
Reckamp, K. L.	2022	Lung-MAP S1800A	≥2	69	Pembrolizumab + Ramucirumab	NR	67	Chemotherapy + Ramucirumab/Chemotherapy	NR	OS
Xiong, A.	2025	HARMONi-2	1	198	Ivonescimab	WT	200	Pembrolizumab	WT	PFS
Lee, K. H.	2025	TASUKI-52/ ONO-4538-52	1	275	Nivolumab + Bevacizumab + Chemotherapy	WT	275	Placebo + Bevacizumab + Chemotherapy	WT	PFS
Park, S.	2024	ATTLAS/ KCSG-LU19-04	≥2	154	Atezolizumab + Bevacizumab + Chemotherapy	95.5% mut	74	Chemotherapy	91.9% mut	PFS
Zhou, C.	2025	IMpower151	1/2	152	Atezolizumab + Bevacizumab + Chemotherapy	52% mut	153	Placebo + Bevacizumab + Chemotherapy	51.6% mut	PFS
Fang, W.	2024	HARMONi-A	≥2	161	Ivonescimab + Chemotherapy	All mut	161	Placebo + Chemotherapy	All mut	PFS
Chen, Z.	2025	HARMONi-6	1	266	Ivonescimab + Chemotherapy	WT	266	Tislelizumab + Chemotherapy	WT	PFS
Wang, L.	2026	ASTRUM-002	1	212	Serplulimab + HLX04 + Chemotherapy	WT	214	Serplulimab + HLX04 Placebo + Chemotherapy	WT	PFS

EGFR, epidermal growth factor receptor; mut, mutant; NR, not reported; WT, wild-type; PFS, progression-free survival; OS, overall survival.

### 3.3 Quality assessment and risk of bias

This meta-analysis used the Cochrane Risk of Bias tool to assess the quality of all included studies. Seven studies implemented blinding for participants and researchers; three studies did not blind outcome assessors; two studies had imbalances in missing participants and reasons between groups; two studies did not report the method of random allocation; and four studies did not report the method of allocation concealment. None of the studies selectively reported results, and it was unclear whether other sources of bias existed. The results of quality assessment are presented in **Supplementary Figures 1**, **2**.

### 3.4 Pooled analysis—results of PFS

**Figure 2:**
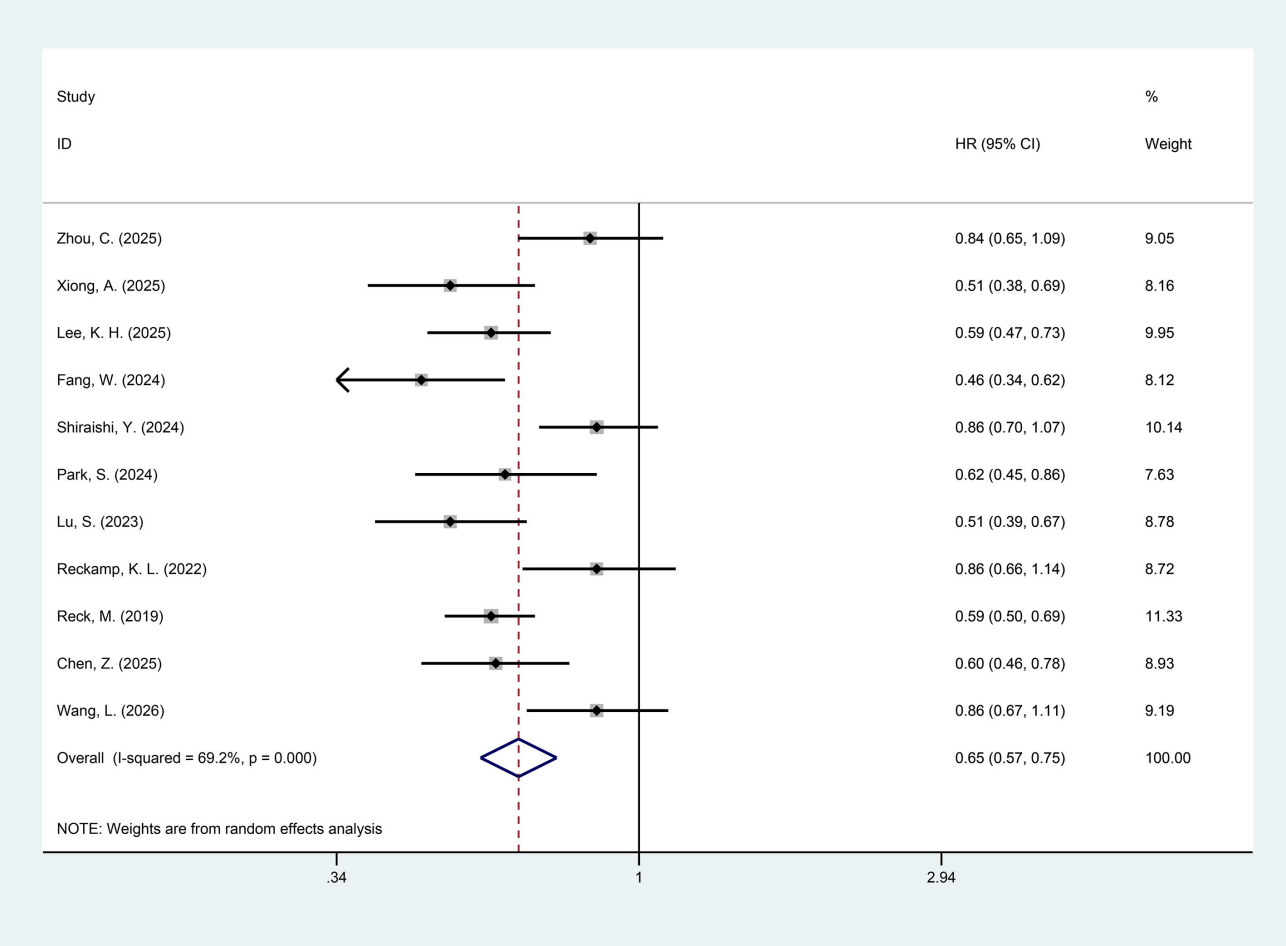
Forest plot of PFS with antibody-based regimens targeting PD-1/PD-L1 and VEGF/VEGFR in advanced or metastatic NSCLC (p<0.001).

A random-effects model was used to pool the PFS data from the eleven included studies. Results indicated that compared with the control group, patients in the intervention group had better PFS (HR = 0.65, 95%CI: 0.57-0.75, p<0.001), as detailed in **Figure 2**. However, the analysis revealed substantial heterogeneity (I²=69.2%), prompting pre-specified subgroup analyses to explore sources of heterogeneity.

Subgroup analysis results are as follows: in the age subgroup, the intervention group demonstrated significantly superior PFS compared with the control group in both patients aged<65 years (HR = 0.59, 95%CI: 0.47-0.74) and those aged≥65 years (HR = 0.68, 95%CI: 0.56-0.82). In the ECOG PS subgroup, both patients with ECOG PS = 0 (HR = 0.62, 95%CI: 0.47-0.82) and those with ECOG PS = 1 (HR = 0.67, 95%CI: 0.59-0.76) had better PFS in the intervention group than in the control group. In the sex subgroup, significant PFS benefits were observed in both male (HR = 0.61, 95%CI: 0.52-0.72) and female patients (HR = 0.74, 95%CI: 0.58-0.95) in the intervention group. In the histology subgroup, the intervention group showed significantly better PFS than the control group in both patients with squamous histology (HR = 0.57, 95%CI: 0.46-0.71) and those with non-squamous histology (HR = 0.67, 95%CI: 0.57-0.79). In the smoking history subgroup, the intervention group demonstrated better PFS compared with the control group in both never-smokers (HR = 0.70, 95%CI: 0.53-0.91) and smokers (HR = 0.68, 95%CI: 0.53-0.88).

Regarding metastatic status, the intervention group demonstrated better PFS compared with the control group regardless of liver metastasis status (with liver metastases: HR = 0.49, 95%CI: 0.39-0.63; without liver metastases: HR = 0.64, 95%CI: 0.50-0.82). The intervention group demonstrated superior PFS compared with the control group regardless of whether patients had brain metastases (with brain metastases: HR = 0.54, 95%CI: 0.43-0.69; without brain metastases: HR = 0.70, 95%CI: 0.59-0.83). In biomarker subgroups, patients in the intervention group demonstrated better PFS than the control group across PD-L1 expression levels by tumor proportion score (TPS)<1% (HR = 0.77, 95%CI: 0.64-0.92), 1%-49% (HR = 0.65, 95%CI: 0.55-0.77), and>50% (HR = 0.65, 95%CI: 0.51-0.83).

In subgroup analyses according to the added treatment component, the intervention group showed better PFS than the control group in all three subgroups: added PD-1/PD-L1 blockade (HR = 0.69, 95%CI: 0.57-0.85), added VEGF/VEGFR blockade (HR = 0.70, 95%CI: 0.54-0.90), and added dual blockade (HR = 0.52, 95%CI: 0.44-0.62). Dual blockade refers to added PD-1/PD-L1 plus VEGF/VEGFR blockade in the intervention group relative to the control group. Detailed data are summarized in **Table 2**.

**Table 2:** Subgroup analysis of progression-free survival.

Subgroup	No. of studies	HR	95% CI	P	Heterogeneity	
					I^2^	P
Age (years)						
<65	8	0.59	(0.47-0.74)	<0.001	69.1%	0.002
≥65	8	0.68	(0.56-0.82)	<0.001	38.4%	0.124
ECOG PS						
0+	10	0.62	(0.47-0.82)	0.001	58.7%	0.010
1	10	0.67	(0.59-0.76)	<0.001	41.2%	0.083
Sex						
Male	9	0.61	(0.52-0.72)	<0.001	52.1%	0.033
Female	8	0.74	(0.58-0.95)	0.018	59.3%	0.016
Histological type						
Squamous	2	0.57	(0.46-0.71)	<0.001	0.0%	0.469
Non-squamous	9	0.67	(0.57-0.79)	<0.001	74.4%	<0.001
Tobacco history						
Never	8	0.70	(0.53-0.91)	0.008	70.0%	0.001
Former or Current	5	0.68	(0.53-0.88)	0.003	52.6%	0.076
Liver metastases						
Yes	8	0.49	(0.39-0.63)	<0.001	0.0%	0.951
No	6	0.64	(0.50-0.82)	<0.001	78.7%	<0.001
Brain metastases						
Yes	8	0.54	(0.43-0.69)	<0.001	26.9%	0.214
No	9	0.70	(0.59-0.83)	<0.001	66.9%	0.002
PD-L1 status						
<1%	7	0.77	(0.64-0.92)	0.004	36.7%	0.149
1-49%	6	0.65	(0.55-0.77)	<0.001	0.0%	0.533
≥50%	8	0.65	(0.51-0.83)	<0.001	35.3%	0.147
Added treatment component						
PD-1/PD-L1 blockade	4	0.69	(0.57-0.85)	<0.001	69.4%	0.020
VEGF/VEGFR blockade	4	0.70	(0.54-0.90)	0.005	74.1%	0.009
Dual blockade	3	0.52	(0.44-0.62)	<0.001	0.0%	0.408

ECOG PS, Eastern Cooperative Oncology Group performance status; PD-L1, programmed death ligand 1; PD-1, programmed cell death protein-1; VEGF, vascular endothelial growth factor; VEGFR, vascular endothelial growth factor receptor; HR, hazard ratio; CI, confidence interval.

**Figure 3:**
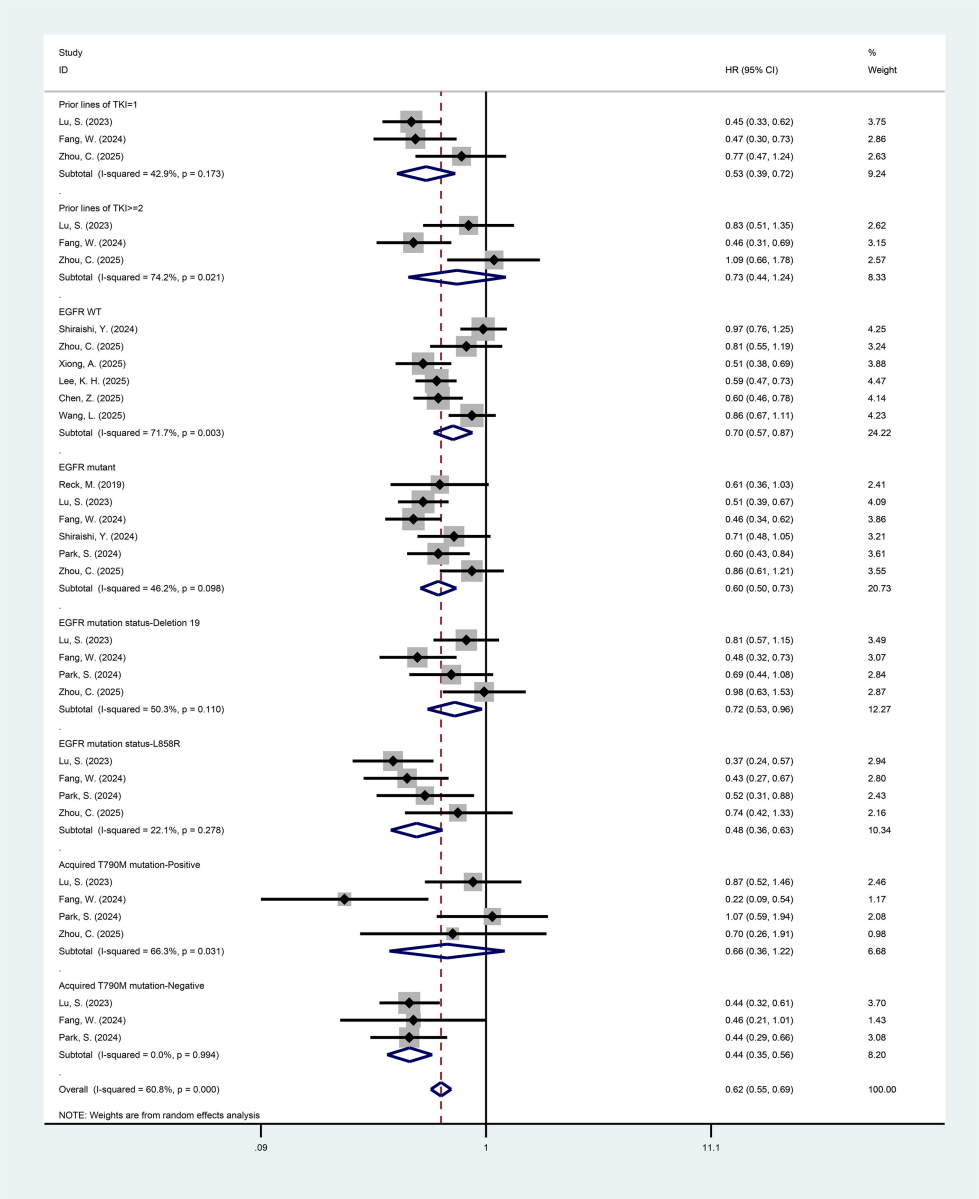
Forest plot of EGFR-related subgroup analyses for PFS.

Based on prior lines of EGFR-TKI therapy, patients in the intervention group who had received only first-line TKI treatment had better PFS than the control group (HR = 0.53, 95%CI: 0.39-0.72), whereas those receiving second-line or later therapy showed no significant difference between the two groups (HR = 0.73, 95%CI: 0.44-1.24). In the EGFR mutation status subgroup, both wild-type patients (HR = 0.70, 95%CI: 0.57-0.87) and EGFR-mutant patients (HR = 0.60, 95%CI: 0.50-0.73) in the intervention group demonstrated better PFS compared with the control group. Specifically, among patients with EGFR exon 19 deletions (HR = 0.72, 95%CI: 0.53-0.96) and EGFR L858R mutations (HR = 0.48, 95%CI: 0.36-0.63), the intervention group demonstrated better PFS compared with the control group. Based on EGFR T790M mutation status, the intervention group had better PFS than the control group in T790M-negative patients (HR = 0.44, 95%CI: 0.35-0.56), whereas no statistically significant difference was observed in T790M-positive patients (HR = 0.66, 95%CI: 0.36-1.22) between the two groups. The relevant content is presented in **Figure 3**.

### 3.5 Pooled analysis—results of OS

**Figure 4:**
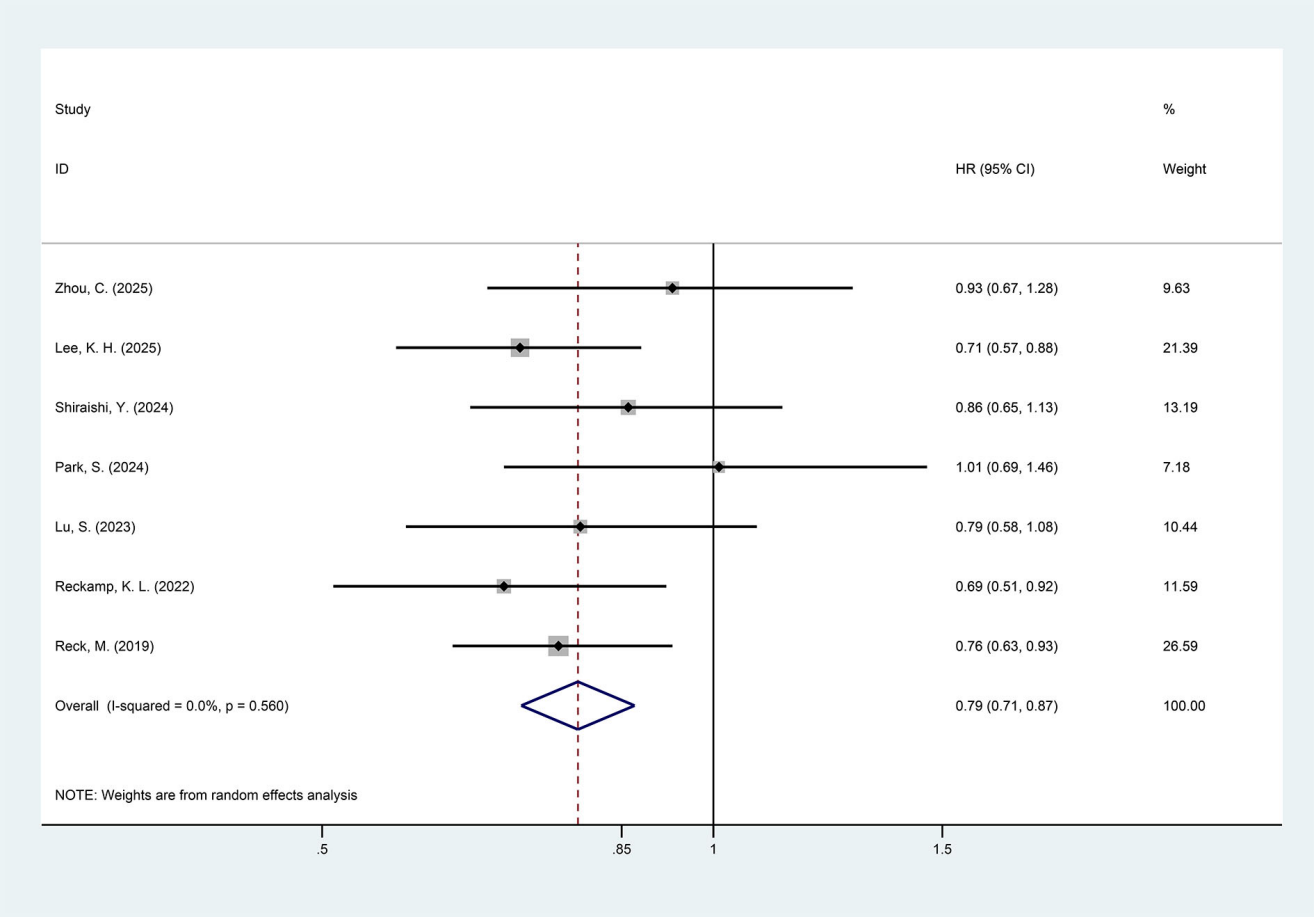
Forest plot of OS with antibody-based regimens targeting PD-1/PD-L1 and VEGF/VEGFR in advanced or metastatic NSCLC (p<0.001).

A pooled analysis of seven studies reporting OS demonstrated that the intervention group achieved a significant improvement in OS compared with the control group (HR = 0.79, 95%CI: 0.71-0.87, p<0.001), with negligible heterogeneity (I²=0%), as shown in **Figure 4**.

The subgroup analysis was conducted as pre-specified, with the following results: in the age subgroup, the OS difference between the intervention and control groups was not statistically significant for neither patients aged<65 years (HR = 0.91, 95%CI: 0.72-1.15) nor those aged≥65 years (HR = 0.73, 95%CI: 0.50-1.09). In the ECOG PS subgroup, the OS difference between the two groups was not statistically significant for patients with ECOG PS = 0 (HR = 0.82, 95%CI: 0.63-1.06); however, patients with ECOG PS = 1 (HR = 0.80, 95%CI: 0.70-0.92) demonstrated a significant OS benefit in the intervention group compared with the control group. In the sex subgroup, the intervention group demonstrated better OS than the control group in male patients (HR = 0.80, 95%CI: 0.68-0.95), whereas no significant difference was observed in female patients (HR = 0.92, 95%CI: 0.74-1.14) between both groups. In the tobacco history subgroup, no statistically significant difference was observed between both groups for non-smokers (HR = 0.90, 95%CI: 0.64-1.25), however, for smokers (HR = 0.82, 95%CI: 0.67-0.99), the intervention group demonstrated superior OS compared with the control group. Regarding metastasis status, among patients with liver metastases (HR = 0.57, 95%CI: 0.40-0.81), the intervention group demonstrated superior OS compared with the control group. In contrast, among patients without liver metastases (HR = 0.92, 95%CI: 0.74-1.15), no statistically significant difference was observed between the intervention and control groups. Regardless of whether patients had brain metastases (HR = 0.79, 95%CI: 0.58-1.08) or did not have brain metastases (HR = 0.87, 95%CI: 0.69-1.08), the OS difference between the intervention and control groups was not statistically significant. Regarding PD-L1 expression by TPS, no statistically significant difference was observed in patients with PD-L1 TPS<1% (HR = 0.84, 95%CI: 0.70-1.02). In patients with PD-L1 TPS 1%-49%, the intervention group showed better OS than the control group (HR = 0.67, 95%CI: 0.51-0.90). However, in the PD-L1 TPS≥50% subgroup, the OS result did not reach statistical significance (HR = 0.77, 95%CI: 0.59-1.01, P = 0.055).

In subgroup analyses according to the added treatment component, the intervention group showed better OS than the control group in the added PD-1/PD-L1 blockade subgroup (HR = 0.75, 95%CI: 0.67-0.85). However, in the dual blockade subgroup, no statistically significant OS difference was observed between the intervention and control groups (HR = 0.87, 95%CI: 0.69-1.11). Detailed results for these subgroup analyses are presented in **Table 3**.

**Table 3:** Subgroup analysis of overall survival. .

Subgroup	No. of studies	HR	95% CI	P	Heterogeneity	
					I^2^	P
Age (years)						
<65	3	0.91	(0.72-1.15)	0.441	22.3%	0.276
≥65	2	0.73	(0.50-1.09)	0.121	0.0%	0.684
ECOG PS						
0	5	0.82	(0.63-1.06)	0.135	13.5%	0.328
1	5	0.80	(0.70-0.92)	0.002	0.0%	0.838
Sex						
Male	4	0.80	(0.68-0.95)	0.010	0.0%	0.695
Female	4	0.92	(0.74-1.14)	0.421	0.0%	0.655
Tobacco history						
Never	4	0.90	(0.64-1.25)	0.516	43.1%	0.153
Former or Current	3	0.82	(0.67-0.99)	0.039	15.0%	0.308
Liver metastases						
Yes	3	0.57	(0.40-0.81)	0.002	0.0%	0.388
No	2	0.92	(0.74-1.15)	0.477	0.0%	0.383
Brain metastases						
Yes	4	0.79	(0.58-1.08)	0.143	0.0%	0.942
No	4	0.87	(0.69-1.08)	0.211	45.2%	0.140
PD-L1 status						
<1%	5	0.84	(0.70-1.02)	0.074	0.0%	0.817
1-49%	3	0.67	(0.51-0.90)	0.007	7.0%	0.341
≥50%	5	0.77	(0.59-1.01)	0.055	3.1%	0.389
Added treatment component						
PD-1/PD-L1 blockade	4	0.75	(0.67-0.85)	<0.001	0.0%	0.520
Dual blockade	2	0.87	(0.69-1.11)	0.267	0.0%	0.323

ECOG PS, Eastern Cooperative Oncology Group performance status; PD-L1, programmed death ligand 1; PD-1, programmed cell death protein-1; HR, hazard ratio; CI, confidence interval.

**Figure 5:**
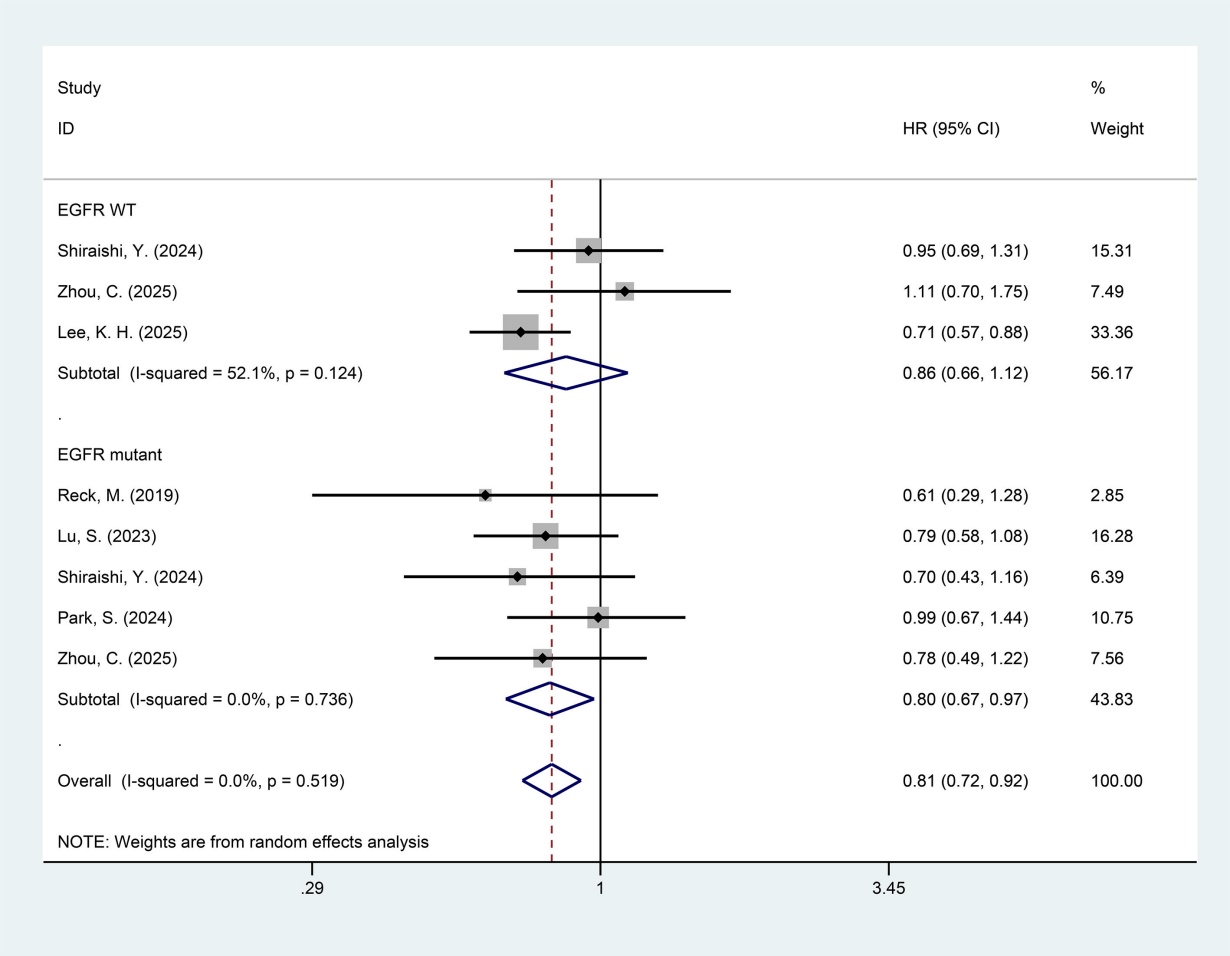
Forest plot of EGFR-related subgroup analyses for OS.meta-analysis.

In the EGFR mutation status subgroup, among EGFR-mutated patients (HR = 0.80, 95%CI: 0.67-0.97), the intervention group had superior OS compared with the control group, while no statistically significant difference was observed between the intervention group and the control group in EGFR wild-type patients (HR = 0.86, 95%CI: 0.66-1.12). The relevant content is presented in **Figure 5**.

### 3.6 Publication bias and sensitivity analysis

To assess the reliability of our findings, this analysis conducted publication bias testing and sensitivity analyses. For PFS, the funnel plot appeared largely symmetrical, and Begg’s test did not detect significant publication bias (p=0.276), as shown in **Supplementary Figure 3**. Sensitivity analysis was performed by sequentially excluding each study, and the pooled HR and 95%CI remained stable without substantial changes, supporting the robustness of the PFS result, as shown in **Supplementary Figure 4**. For OS, the funnel plot appeared largely symmetrical, and Begg’s test did not detect significant publication bias (p=0.072), as shown in **Supplementary Figure 5**. Similarly, sequential removal of individual studies did not materially change the pooled HR or 95%CI for OS, indicating that the OS result was also stable, as shown in **Supplementary Figure 6**.

## 4 Discussion

This meta-analysis demonstrated that, in patients with advanced or metastatic NSCLC, antibody-based regimens targeting PD-1/PD-L1 and VEGF/VEGFR, including conventional PD-1/PD-L1 inhibitors combined with anti-angiogenic monoclonal antibodies and PD-1/VEGF bispecific antibody, with or without chemotherapy, significantly prolonged both PFS and OS. In the predefined subgroup analyses, more favorable PFS estimates were observed in men, patients with squamous histology, those with liver metastases, those with higher PD-L1 expression, and those with specific EGFR mutational features (L858R mutation, only one prior line of EGFR-TKI therapy, and no concomitant T790M mutation); whereas more favorable OS estimates were observed in patients with ECOG PS = 1, men, smokers, those with liver metastases, those with PD-L1 TPS 1%-49%, and those with EGFR mutations. In addition, subgroup analyses stratified by the added treatment component were performed to further examine the influence of different control regimens on the pooled estimates. Overall, these subgroup findings should be interpreted as hypothesis-generating rather than definitive evidence of predictive effects.

To better understand these overall and subgroup survival findings, we considered the results from two perspectives. First, the complementary interaction between PD-1/PD-L1 blockade and VEGF/VEGFR inhibition may help explain the overall benefit observed in this meta-analysis. Second, clinical and molecular differences among patients may help explain the variation in treatment estimates across subgroups.

The observed survival improvement is biologically plausible given the complementary effects of PD-1/PD-L1 blockade and VEGF/VEGFR inhibition. In conventional combination regimens, PD-1/PD-L1 inhibitors restore antitumor immunity by blocking PD-1/PD-L1 signaling and reversing T-cell functional suppression. Anti-angiogenic monoclonal antibodies (e.g., bevacizumab targeting VEGF and ramucirumab targeting VEGFR-2) inhibit VEGF-driven angiogenesis and the associated immunosuppressive effects. A major contributor to the synergy is the ability of anti-angiogenic monoclonal antibodies to attenuate VEGF/VEGFR-mediated immunosuppression. VEGF exerts multifaceted immunosuppressive effects within the tumor microenvironment, as outlined below: (1) VEGF promotes recruitment and expansion of immunosuppressive cell populations, including regulatory T cells (Tregs) (29), myeloid-derived suppressor cells (MDSCs) (30, 31), and immunosuppressive tumor-associated macrophage (TAM) phenotypes (32). (2) VEGF weakens antitumor T-cell responses through both indirect and direct pathways. Indirectly, it impairs DC maturation and antigen presentation, thereby limiting T-cell priming (33, 34). Directly, it suppresses T-cell proliferation and cytotoxicity and is accompanied by upregulation of inhibitory checkpoint molecules (e.g., PD-1, PD-L1 and cytotoxic T lymphocyte antigen 4(CTLA-4)) (35). In addition, VEGF may also affect upstream T-cell generation by limiting hematopoietic stem cell differentiation toward the CD4^+^CD8^+^ T-cell (36). (3) VEGF downregulates endothelial adhesion molecules (e.g., intercellular adhesion molecule 1 (ICAM-1) and vascular cell adhesion molecule 1 (VCAM-1)) and maintains abnormal vascular structure and function, restricting immune-cell adhesion, transendothelial migration, and intratumoral infiltration (37–39). By disrupting VEGF/VEGFR signaling, anti-angiogenic monoclonal antibodies alleviate these immunosuppressive mechanisms, thereby restoring a more immune-permissive tumor microenvironment that enhances the efficacy of PD-1/PD-L1 inhibitors. This mechanistic rationale also applies to PD-1/VEGF bispecific antibodies, such as ivonescimab, which integrate PD-1 blockade and VEGF inhibition within a single antibody-based molecule. However, because most included regimens were conventional combinations of PD-1/PD-L1 inhibitors and anti-angiogenic monoclonal antibodies, the mechanisms discussed here primarily refer to these combination regimens.

Another major contributor to this synergy is vascular normalization induced by VEGF/VEGFR inhibition, through which anti-angiogenic monoclonal antibodies may improve intratumoral drug delivery and immune-effector access: (1) optimized vascular permeability and perfusion can increase the intratumoral delivery and distribution efficiency of therapeutic macromolecules, including PD-1/PD-L1 antibodies (40, 41); (2) improved oxygenation and reduced interstitial pressure can decrease hypoxia-related immunosuppressive signaling (42, 43)and promote effector T-cell infiltration and sustained activity in tumors (42). Taken together, these immune-vascular effects offer a plausible biological explanation for why PD-1/PD-L1 inhibitors may perform better when paired with anti-angiogenic monoclonal antibodies in NSCLC. Importantly, such a strategy is clinically feasible and has precedent. For example, based on IMpower150, bevacizumab plus atezolizumab and chemotherapy has been approved as first-line therapy for advanced non-squamous NSCLC without targetable driver alterations.

In our predefined subgroup analyses, more favorable treatment-effect estimates were observed in men, current or former smokers, patients with squamous histology, those with ECOG PS = 1, and those with higher PD-L1 expression. These subgroup patterns should be viewed as exploratory and hypothesis-generating. Biologically, these features may collectively reflect a tobacco-exposure-enriched clinical-biological phenotype rather than isolated determinants of treatment response.

Smoking-related mutagenesis and inflammation are associated with higher tumor mutational burden (TMB) and increased PD-L1 expression, which in turn correlate with greater benefit from PD-1/PD-L1 blockade (44–47). In addition, tobacco-related carcinogens (e.g., nicotine) can upregulate VEGF and its receptors through the α7 nicotinic acetylcholine receptor (α7-nAChR) pathway (48), thereby strengthening pro-angiogenic signaling and promoting an immunosuppressive tumor microenvironment (49). In the setting of smoking-associated PD-L1 upregulation together with enhanced VEGF signaling, two treatment-relevant features often coexist. First, high PD-L1 expression in some patients may reflect IFN-γ–driven adaptive immune resistance and the presence of tumor-infiltrating lymphocytes (TILs), suggesting that PD-1/PD-L1 blockade can reinvigorate pre-existing antitumor T-cell activity by releasing checkpoint restraint (50). Second, activation of the VEGF axis indicates concurrent angiogenic dependence and VEGF-associated immunosuppression, supporting the rationale for combined PD-1/PD-L1 blockade and VEGF/VEGFR inhibition. Consistent with the mechanisms described above, VEGF/VEGFR inhibition can inhibit angiogenesis and partially alleviate related immunosuppression, thereby potentially enhancing the antitumor activity of PD-1/PD-L1 blockade (14, 49, 51).

In addition, the clustering of this phenotype with squamous histology, male, and ECOG PS = 1 is epidemiologically and biologically consistent. Squamous carcinoma is strongly associated with smoking (52), often accompanied by higher PD-L1 expression (53, 54) and higher TMB (53), and it may show more active VEGF signaling (55, 56). Enrichment in men likely reflects higher historical tobacco exposure (57).

Smoking may be more common among patients with ECOG PS = 1. A prior study stratified by World Health Organization (WHO) PS (a score similar to and interchangeable with ECOG PS) likewise observed a higher smoking proportion in the PS = 1 group (58). Taken together, these findings provide a biologically plausible rationale for antibody-based regimens targeting PD-1/PD-L1 and VEGF/VEGFR, particularly conventional PD-1/PD-L1 inhibitor plus anti-angiogenic monoclonal antibody combinations, in smoking-related phenotypes, and suggest that future work could further refine patient stratification and validate this phenotype prospectively.

In addition to smoking-associated tumor biology, organ-specific metastatic sites can further shape the immune microenvironment and may contribute to variation in treatment estimates. In our subgroup analyses, a favorable pattern was observed in patients with liver metastases receiving antibody-based regimens targeting PD-1/PD-L1 and VEGF/VEGFR. Several studies have reported liver metastases as a predictor of poorer outcomes with PD-1/PD-L1 inhibitor monotherapy (59, 60). This is consistent with the organ-specific immunoregulatory functions of the liver (61), which can both impair CD8^+^ T-cell function (62) and abnormally activate Tregs (63), ultimately leading to systemic failure of antitumor immunity. Against this background, VEGF/VEGFR inhibition may be particularly relevant because it can remodel the immunosuppressive tumor milieu and facilitate a more immune-permissive environment for PD-1/PD-L1 blockade, as discussed above (61, 64, 65). Collectively, these considerations provide a biological rationale for the favorable estimate observed in the liver-metastasis subgroup. Notably, exploratory subgroup analyses from the APPLE study also suggested a greater benefit of PD-1/PD-L1 inhibitors plus anti-angiogenic monoclonal antibodies in patients with liver metastases than in those without liver involvement (21).

In addition to organ metastasis, driver-gene background and treatment sequence may also influence immune status and angiogenic dependence, thereby contributing to the observed subgroup patterns. Our meta-analysis found that EGFR-mutant patients showed more favorable survival estimates than EGFR wild-type patients, primarily in trials evaluating PD-1/PD-L1 inhibitors plus anti-angiogenic monoclonal antibodies with chemotherapy. Although basic studies suggest that EGFR pathway activation can directly upregulate PD-L1 through multiple mechanisms and promote immune escape at the cellular and molecular levels (66, 67), EGFR-mutant tumors at the population level often exhibit lower TMB and a relatively cold tumor microenvironment, resulting in lower PD-L1 expression and poor responses to PD-1/PD-L1 monotherapy (68). According to National Comprehensive Cancer Network (NCCN) guidelines, EGFR-TKIs remain the standard first-line therapy in this setting (69). Real-world evidence further supports the clinical dominance of EGFR-TKI-based first-line management: a nationwide Greek e-prescription study showed rapid replacement of earlier EGFR-TKIs by newer-generation agents, with osimertinib becoming the most commonly prescribed first-line EGFR-TKI, highlighting the increasing clinical relevance of effective post-TKI treatment strategies (70). Notably, emerging studies indicate that EGFR-TKI treatment itself can dynamically remodel the tumor immune microenvironment, potentially shifting it from an immunosuppressive to a more immunosupportive state, thereby creating a theoretical opportunity for PD-1/PD-L1 blockade-based strategies after EGFR-TKI resistance (71). Moreover, EGFR-mutant tumors often show higher VEGF expression, and after EGFR-TKI resistance, VEGF signaling may become a key alternative pathway sustaining tumor growth (72). Consequently, anti-angiogenic monoclonal antibodies (e.g., bevacizumab) combined with chemotherapy have become a standard of care after EGFR-TKI resistance (69). In the RCTs included in our meta-analysis, most EGFR-mutant patients were enrolled after EGFR-TKI failure. In this clinical context, the potential rationale for the triplet regimen may lie in simultaneously addressing two biological features that are common after resistance: (1) checkpoint-mediated suppression of T-cell function, which may be relieved by PD-1/PD-L1 blockade; and (2) persistent VEGF/VEGFR-related angiogenic drive, which can be inhibited by anti-angiogenic antibodies while also mitigating VEGF-associated immunosuppression. The convergence of these mechanisms may provide a plausible explanation for the favorable efficacy signal observed with the triplet regimen after EGFR-TKI resistance, as supported by our findings and prior evidence (73). It should be noted that, across the three EGFR-related subgroup analyses, the intervention arms of all included RCTs used the triplet regimen of PD-1/PD-L1 inhibitors plus anti-angiogenic antibodies plus chemotherapy in our meta-analysis (18, 22, 24, 26). Thus, our inferences regarding EGFR subgroups are primarily grounded in this triplet-therapy evidence framework.

To explore which EGFR-mutant patients might show more favorable outcomes with the triplet regimen, we considered three closely linked variables along a single treatment sequence: EGFR mutation subtype (L858R vs exon 19 deletion), acquired T790M status, and the number of prior EGFR-TKI lines. A previous meta-analysis suggested that, before EGFR-TKI treatment, baseline T790M co-mutation more commonly coexists with EGFR L858R and is less frequent in exon 19 deletion (74). However, after EGFR-TKI exposure and acquired resistance, T790M becomes more common in exon 19 deletion patients (75). This dynamic shift directly influences subsequent treatment pathways. As a key resistance mechanism, T790M usually leads patients who fail first- or second-generation EGFR-TKIs to receive third-generation EGFR-TKIs (e.g., osimertinib). Therefore, T790M-positive patients are more likely to have second-line or later TKI exposure and follow a longer sequence of TKI therapy (76, 77). In contrast, after first-line EGFR-TKI failure, T790M-negative patients often lack a clear basis to continue TKIs, and more commonly move directly into a second-line framework centered on chemotherapy ± anti-angiogenic treatment. As a result, the triplet regimen is more readily considered for these patients in terms of timing and clinical decision-making. Beyond treatment-path differences, T790M status also correlates with microenvironmental features related to immunotherapy sensitivity. Haratani et al. reported that T790M-negative patients have higher PD-L1 expression than T790M-positive patients and are more likely to benefit from nivolumab (78). In T790M-positive patients, fewer tumor-infiltrating T cells (e.g., CD8^+^PD-1^+^ T cells and CD8^+^TIM-3^+^ T cells) have also been observed, suggesting a weaker immune-reactive baseline (79). In addition, clinical management differences may contribute. Because T790M-positive patients often receive longer TKI sequences (including third-generation TKIs), accumulated toxicity may affect tolerance and the feasible intensity of subsequent intensified combination treatment (80, 81).

Based on the relationship among resistance mechanisms, treatment sequencing, and immune microenvironment features, we propose a hypothesis-generating interpretive framework. Within this framework, the observed subgroup pattern may be compatible with a scenario in which the triplet regimen may be more relevant in the second-line setting after first-line EGFR-TKI failure and before entry into multi-line TKI sequencing, a scenario that may more often correspond clinically to patients with L858R mutations and T790M-negative status. By contrast, exon 19 deletion patients are more likely to develop T790M after acquired resistance and thus more often proceed to multi-line sequencing such as third-generation TKIs; the timing and immune baseline at which they receive the triplet regimen may therefore differ from the group described above. The trends observed in our subgroup analyses are consistent with this interpretation, but require prospective validation.

In addition to the clinical and molecular factors discussed above, differences in control regimens may also contribute to clinical heterogeneity and affect the interpretation of the pooled results. To address this issue, subgroup analyses were performed according to the treatment component added in the intervention arm relative to the control arm: added PD-1/PD-L1 blockade, added VEGF/VEGFR blockade, or dual blockade. This approach separated trials according to what was newly added to the intervention arm, thereby making the different control-regimen settings easier to interpret. PFS improvement was observed in all three subgroups, indicating that the direction of the PFS effect remained consistent after these different comparison settings were separated. Therefore, the overall PFS result was not driven only by trials in which the control arm lacked both PD-1/PD-L1 and VEGF/VEGFR blockade, such as chemotherapy-based controls. Nevertheless, the lower HR in the dual blockade subgroup may partly reflect the larger treatment contrast between the intervention and control arms, because the control arms in this subgroup usually contained more basic treatment. OS results were less consistent and were mainly observed in the added PD-1/PD-L1 blockade subgroup, possibly due to fewer OS events, immature follow-up, subsequent therapies, and differences in trial design. Thus, this subgroup analysis helps clarify how different control regimens may influence the pooled results, but the findings should still be interpreted cautiously.

The different patterns observed for PFS and OS also warrant further consideration. Mechanistic complementarity and subgroup-level signals do not necessarily translate into improvements of the same magnitude across all endpoints. This also underscores the need to examine the sources of divergence between PFS and OS. Overall, OS remains the gold standard endpoint for anticancer efficacy, but it usually requires longer follow-up and can be strongly influenced by post-progression therapies. Accordingly, many NSCLC randomized trials use early endpoints such as PFS as primary outcomes to more rapidly reflect direct control of disease progression (82). However, methodological studies have noted that, in NSCLC—particularly in the immunotherapy era—PFS is not a stable surrogate for OS, and improvements in PFS do not necessarily translate proportionally into improvements in OS (83). Consistent with this, our meta-analysis observed a more pronounced benefit for PFS (HR = 0.65), whereas the OS benefit was more conservative (HR = 0.79). Regarding why PFS appears more “prominent,” first, PFS treats all radiographic progression events as equivalent, while different progression patterns may have different implications for subsequent survival (e.g., delaying distant metastasis versus delaying mild local progression may contribute differently to OS). This makes PFS statistically more sensitive, while the “survival weight” of PFS improvement may be heterogeneous (84). Second, even though most included studies used Response Evaluation Criteria in Solid Tumors (RECIST) v1.1, there were still objective differences in PFS measurement, including differences in assessors (some trials used independent review committees such as Independent Radiology Review Committee (IRRC) or Blinded Independent Central Review (BICR), whereas others used investigator-assessed PFS as the primary endpoint) and differences in imaging schedules [e.g., shifting from every 6 weeks to every 9 weeks versus shifting from every 6 weeks to every 12 weeks, as in ORIENT-31 (26), HARMONi-6 (23), ASTRUM-002 (28), and IMpower151 (18)]. Such differences affect when progression is recorded, making PFS more vulnerable to measurement variability and potentially yielding a more prominent pooled effect in meta-analysis. As for why OS appears “less obvious,” first, OS data were immature and/or event numbers were insufficient in some studies at the data cutoff, so true differences may not yet have emerged (e.g., ASTRUM-002 (28), HARMONi-A (24), and IMpower151 (18) noted immature OS or limited follow-up). Second, OS reflects the entire treatment sequence; post-progression therapies (especially subsequent immunotherapy in control arms or treatment crossover) can substantially narrow OS differences between arms. For example, in TASUKI-52 (27), subsequent immunotherapy use was higher in the control arm, and the authors explicitly noted that this might reduce OS differences.

Therefore, the pattern of “more pronounced PFS benefit and more conservative OS benefit” is not contradictory. PFS more sensitively captures the direct effect of the combination on first progression but is more affected by event composition and measurement variability. OS is more dependent on follow-up maturity and is more easily diluted by post-progression treatment. Accordingly, PFS can be interpreted as an earlier efficacy signal, whereas OS should be interpreted more cautiously in light of follow-up and the post-progression treatment landscape.

This study has several limitations. First, the number of included RCTs was limited, and sample sizes were relatively small in some subgroups (particularly EGFR subtype, T790M status, and liver metastases). Thus, subgroup findings may be subject to chance. Some subgroup signals were clearer for PFS but not replicated to the same extent for OS, which may reflect insufficient events, immature follow-up, and dilution by post-progression therapies. In addition, subgroup analyses were primarily exploratory and hypothesis-generating, and should not be regarded as definitive evidence of predictive factors. Second, differences across studies in regimen composition and implementation (use of chemotherapy, type of anti-angiogenic antibody, imaging schedules, and PFS assessment systems) may introduce heterogeneity and affect endpoint comparability. As more RCTs are completed and data mature, these conclusions should be updated. Third, because only 11 studies for PFS and 7 studies for OS were included, the funnel plots and Begg’s tests may have had limited power to detect potential publication bias. Fourth, the control regimens varied across the included trials, ranging from chemotherapy-based controls to regimens containing PD-1/PD-L1 blockade or VEGF/VEGFR inhibition. Although we performed subgroup analyses according to the added treatment component to improve interpretability, this approach could not fully eliminate the clinical heterogeneity introduced by different control-arm regimens. Therefore, the pooled estimates should be interpreted cautiously.

## 5 Conclusion

This meta-analysis indicates that, in advanced or metastatic NSCLC, antibody-based regimens targeting PD-1/PD-L1 and VEGF/VEGFR, including conventional PD-1/PD-L1 inhibitors combined with anti-angiogenic monoclonal antibodies and PD-1/VEGF bispecific antibodies, with or without chemotherapy, are associated with statistically significant improvements in both PFS and OS. In predefined subgroup analyses, favorable PFS estimates were observed in men, patients with squamous histology, liver metastases, higher PD-L1 expression, and specific EGFR-related treatment-sequence features (e.g., L858R mutation, prior exposure to first-line EGFR-TKI only, and T790M-negative status), whereas favorable OS estimates were observed in patients with ECOG PS = 1, men, smokers, those with liver metastases, PD-L1 TPS 1%-49%, and EGFR-mutant disease. Overall, these findings support antibody-based regimens targeting PD-1/PD-L1 and VEGF/VEGFR as potential therapeutic options for advanced or metastatic NSCLC, while the observed subgroup patterns warrant further validation before being incorporated into treatment decision-making.

## Supplementary material

Supplementary Figure 1 Risk of bias assessment in included studies.

Supplementary Figure 2 Summary of risk of bias across studies.

Supplementary Figure 3 Begg’s funnel plot showing publication bias of PFS (p=0.276).

Supplementary Figure 4 Plot of sensitivity analysis of PFS.

Supplementary Figure 5 Begg’s funnel plot showing publication bias of OS (p=0.072).

Supplementary Figure 6 Plot of sensitivity analysis of OS.
