# Supplementary figures and images for: Antibody-based regimens targeting PD-1/PD-L1 and VEGF/VEGFR in advanced or metastatic NSCLC: a meta-analysis of RCTs
Source: Front Immunol. 2026 Jun 9;17:1847913. doi: 10.3389/fimmu.2026.1847913 (PMC13287054; doi:10.3389/fimmu.2026.1847913)

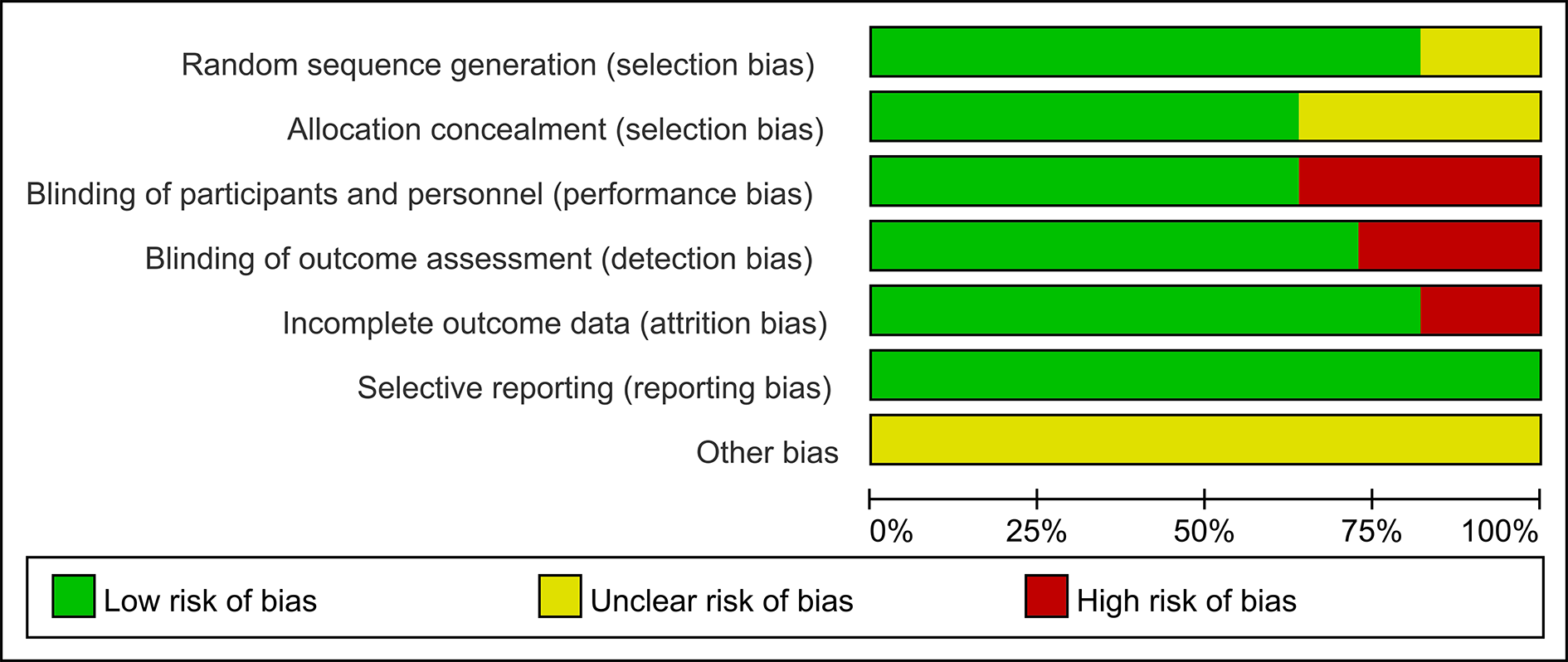

Supplement: Supplementary Figure 1 — Risk of bias assessment in included studies. [file Image1.tif]

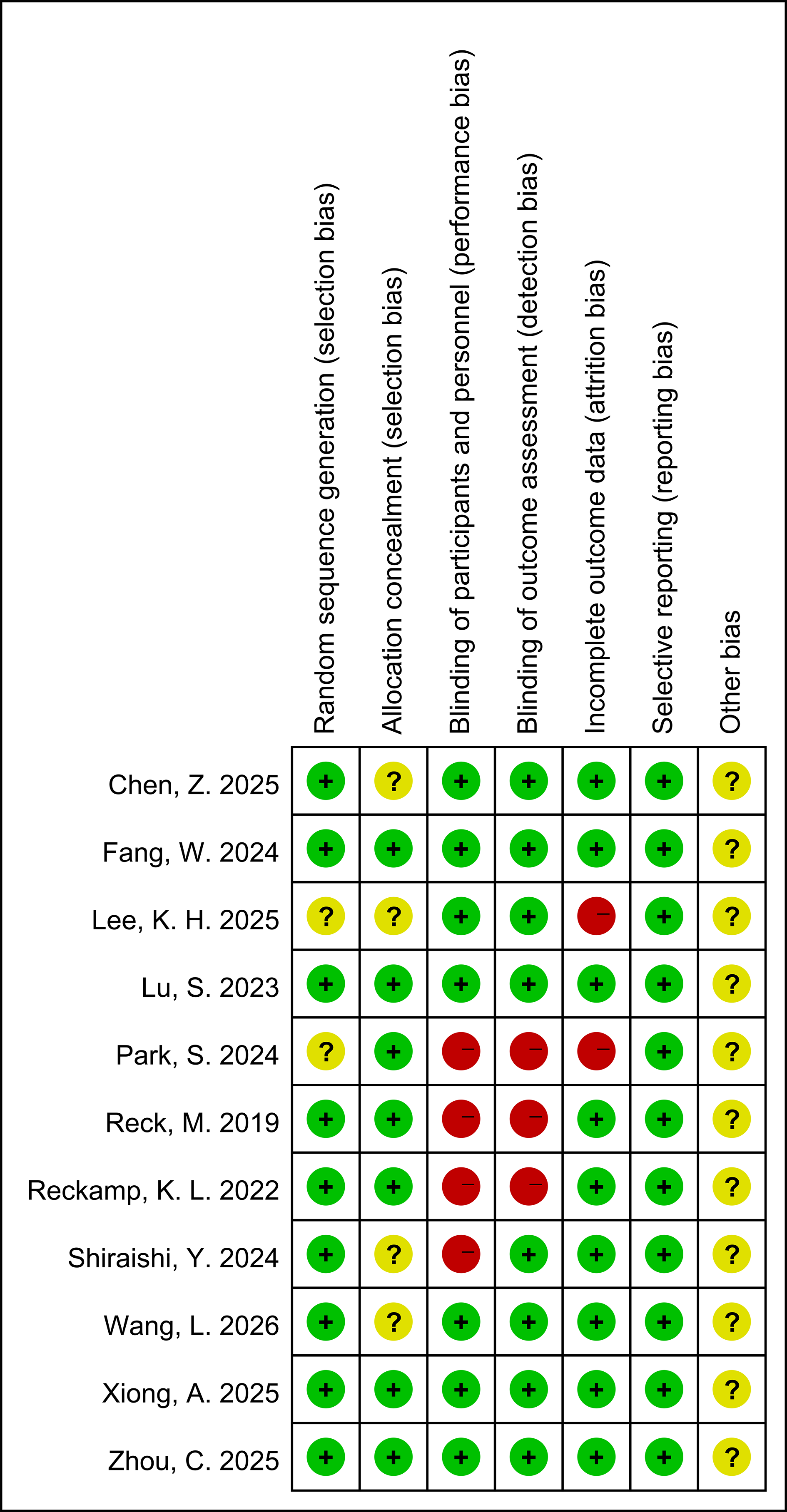

Supplement: Supplementary Figure 2 — Summary of risk of bias across studies. [file Image2.tif]

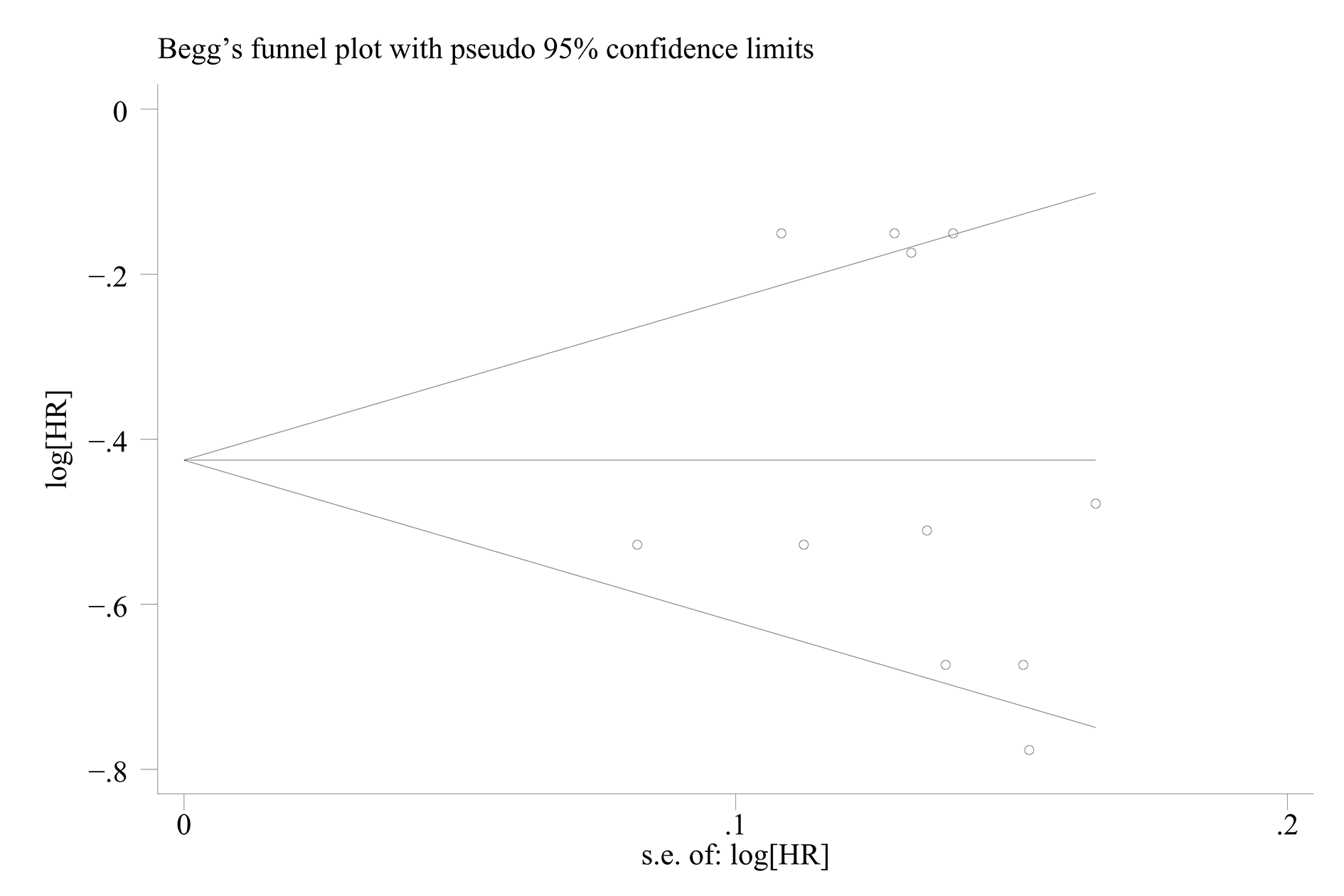

Supplement: Supplementary Figure 3 — Begg’s funnel plot showing publication bias of PFS (p=0.276). [file Image3.tif]

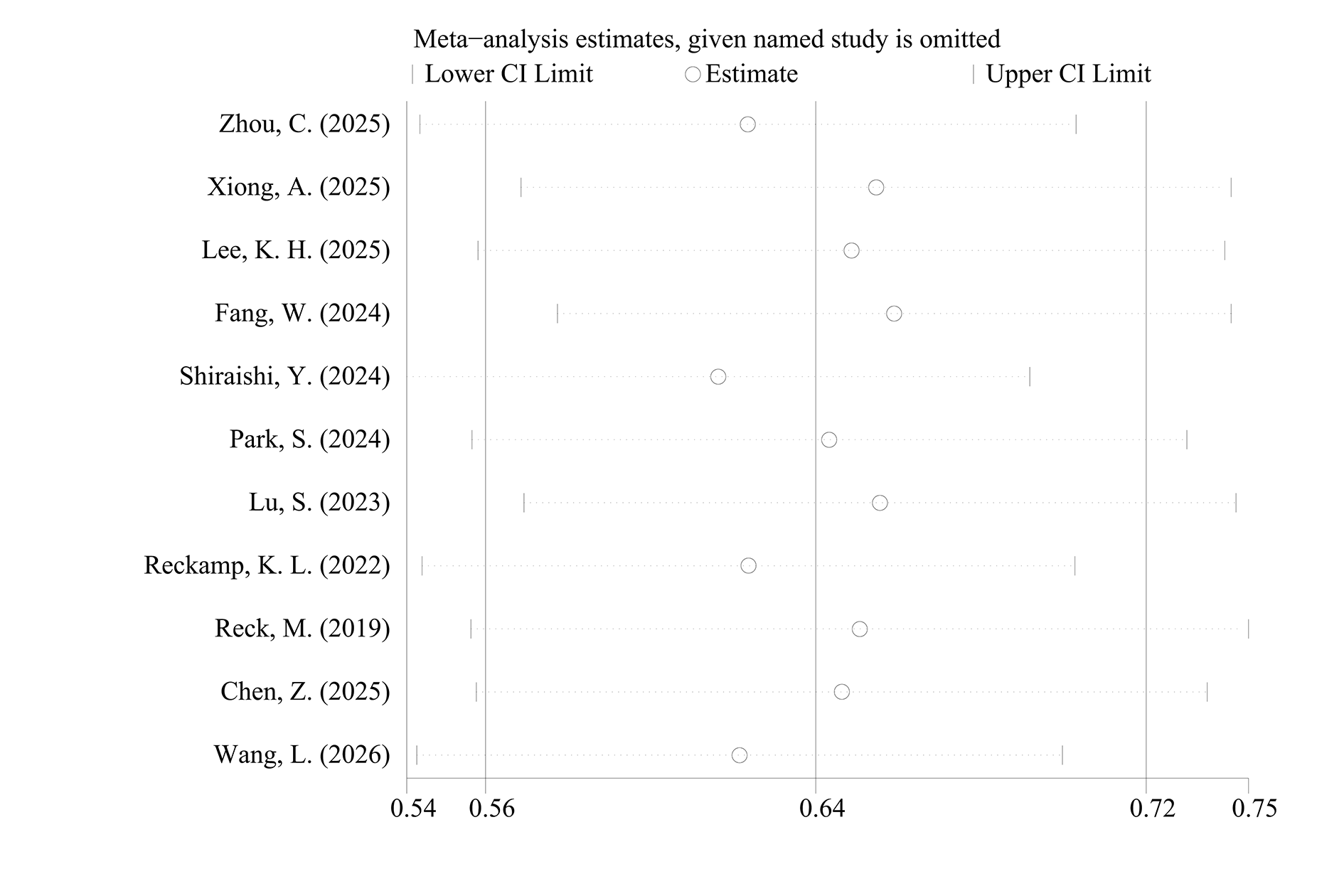

Supplement: Supplementary Figure 4 — Plot of sensitivity analysis of PFS. [file Image4.tif]

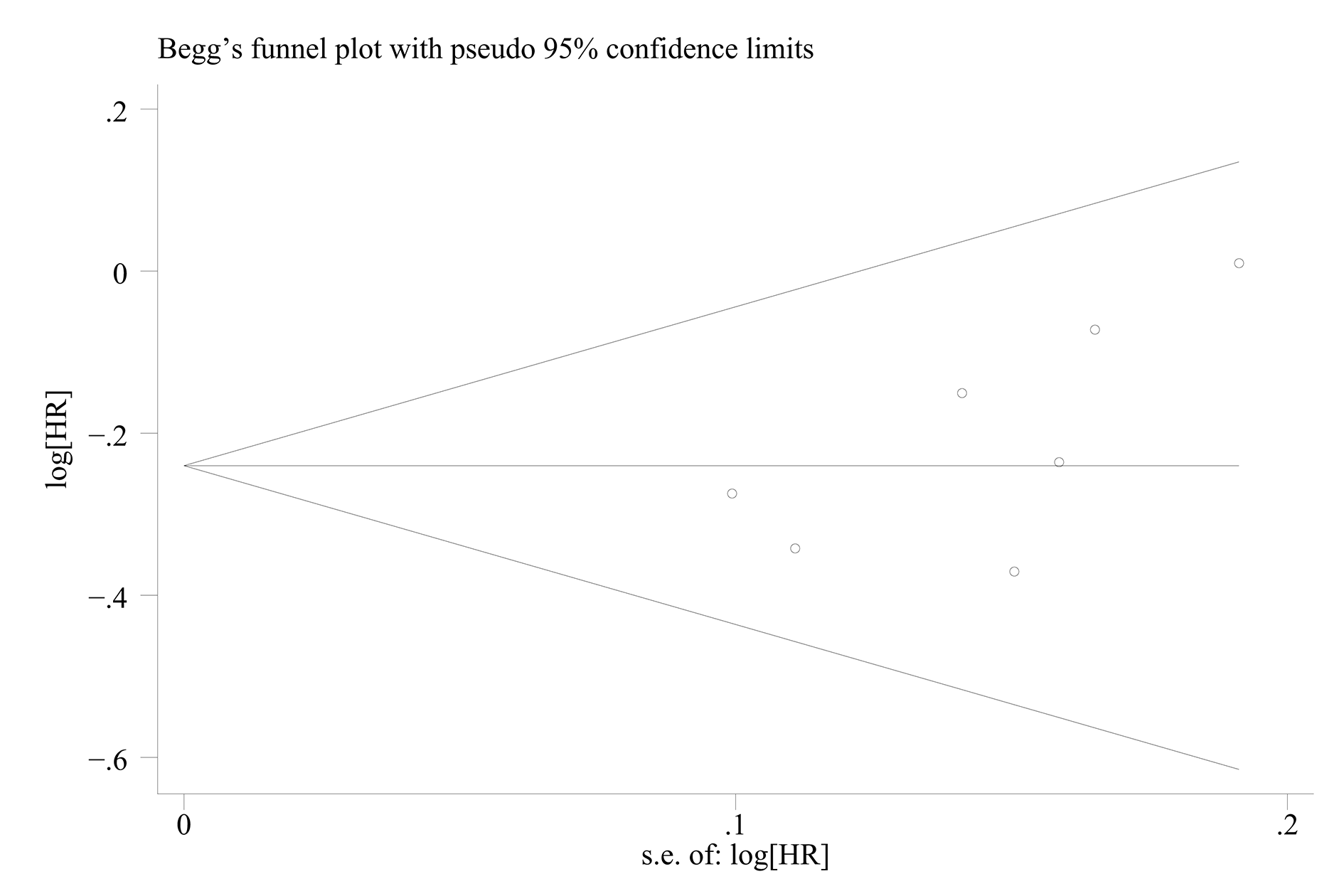

Supplement: Supplementary Figure 5 — Begg’s funnel plot showing publication bias of OS (p=0.072). [file Image5.tif]

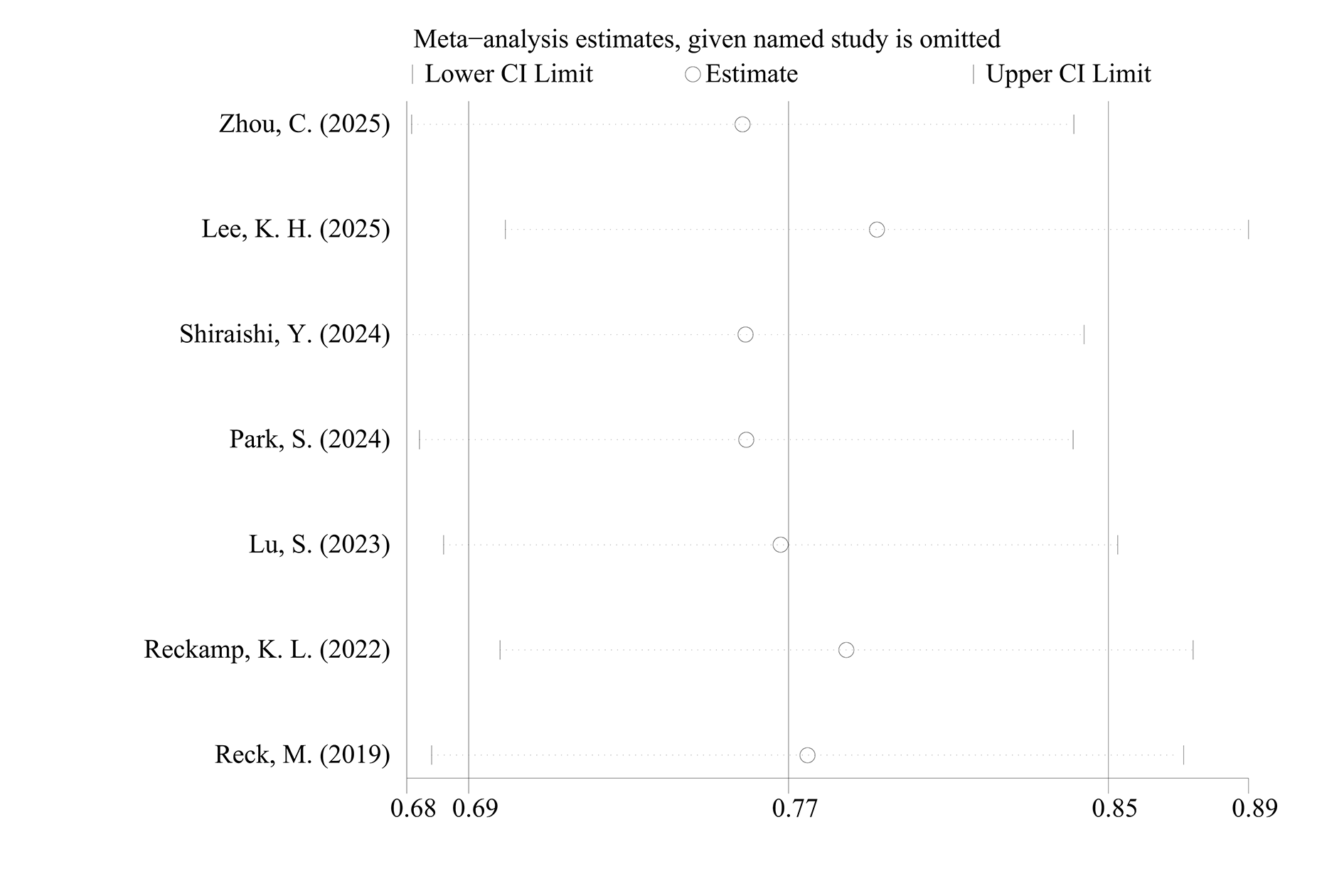

Supplement: Supplementary Figure 6 — Plot of sensitivity analysis of OS. [file Image6.tif]
