# Supplementary material for: Antibody-based regimens targeting PD-1/PD-L1 and VEGF/VEGFR in advanced or metastatic NSCLC: a meta-analysis of RCTs
Source: Front Immunol. 2026 Jun 9;17:1847913. doi: 10.3389/fimmu.2026.1847913 (PMC13287054; doi:10.3389/fimmu.2026.1847913)
Supplement: Supplementary file 7 [file Table1.doc]

**Supplementary Table 1. Characteristics of the randomized controlled trials included in this meta-analysis.**

| Author | Year | Country | Clinical trial  number | Recruiting  patient time | Median Follow-Up (Months) | Histology | Intervene | | Control | |
| --- | --- | --- | --- | --- | --- | --- | --- | --- | --- | --- |
|  |  |  |  |  |  |  | EGFR mutation subtype, n/N | Acquired T790M mutation, n | EGFR mutation subtype, n/N | Acquired T790M mutation, n |
| Lu, S. | 2023 | China | NCT03802240 | 2019.07-2022.03 | 12.9-14.4 | NS-NSCLC | Deletion 19: 80/158  L858R: 70/158 | 40 | Deletion 19: 88/160  L858R: 61/160 | 40 |
| Reck, M. | 2019 | Multi-countries | NCT02366143 | 2015.03-2016.12 | 19.6-19.7 | NS-NSCLC | Deletion 19 + L858R: 26/34 | 1 | Deletion 19 + L858R: 32/45 | 2 |
| Shiraishi, Y. | 2024 | Japan | jRCT2080224500 | 2019.01-2020.08 | NR | NS-NSCLC | Deletion 19: 29/54  L858R: 22/54 | NR | Deletion 19: 28/59  L858R: 24/59 | NR |
| Reckamp, K. L. | 2022 | United States | NCT03971474 | 2019.05-2020.11 | 17.9 | NSCLC | NR | NR | NR | NR |
| Xiong, A. | 2025 | China | NCT05499390 | 2022.11-2023.08 | 8.7 | NS-NSCLC | 0 | 0 | 0 | 0 |
| Lee, K. H. | 2025 | Multi-countries | NCT03117049 | 2017.06-2019.07 | 13.7 | NS-NSCLC | 0 | 0 | 0 | 0 |
| Park, S. | 2024 | Republic of Korea | NCT03991403 | 2019.08-2022.03 | 26.1 | NSCLC | Deletion 19: 70/147  L858R: 75/147 | 51 | Deletion 19: 42/68  L858R: 25/68 | 20 |
| Zhou, C. | 2025 | China | NCT04194203 | 2020.04-2022.03 | 14.0 | NS-NSCLC | Deletion 19: 50/79  L858R: 26/79 | 14 | Deletion 19: 46/79  L858R: 31/79 | 8 |
| Fang, W. | 2024 | China | NCT05184712 | 2022.01-2022.11 | 7.9 | NS-NSCLC | Deletion 19: 92/161  L858R: 60/161 | 26 | Deletion 19: 78/161  L858R: 78/161 | 18 |
| Chen, Z. | 2025 | China | NCT05840016 | 2023.08-2025.01 | 10.3 | Sq-NSCLC | 0 | 0 | 0 | 0 |
| Wang, L. | 2026 | China | NCT03952403 | 2019.11-2022.06 | 23.1-23.4 | NS-NSCLC | 0 | 0 | 0 | 0 |

For EGFR mutation subtype, N is the number of patients with available EGFR subtype data. Abbreviations: NR, nor reported; NS, non-squamous; Sq, squamous; NSCLC, non-small cell lung cancer; EGFR: epidermal growth factor receptor.
