# Supplementary material for: Antibody-based regimens targeting PD-1/PD-L1 and VEGF/VEGFR in advanced or metastatic NSCLC: a meta-analysis of RCTs
Source: Front Immunol. 2026 Jun 9;17:1847913. doi: 10.3389/fimmu.2026.1847913 (PMC13287054; doi:10.3389/fimmu.2026.1847913)
Supplement: Supplementary file 8 [file Table2.docx]

**Supplementary Search Strategy**

**Pubmed**

| Search number | Search Details | Results |
| --- | --- | --- |
| 1 | ((((((((((((((((((((((((((((((((((((((((Immune Checkpoint Inhibitors[MeSH Terms]) OR (Checkpoint Inhibitors, Immune)) OR (Immune Checkpoint Blockers)) OR (Checkpoint Blockers, Immune)) OR (Immune Checkpoint Inhibitor)) OR (Checkpoint Inhibitor, Immune)) OR (PD-1 Inhibitors)) OR (PD 1 Inhibitors)) OR (Programmed Cell Death Protein 1 Inhibitor)) OR (Programmed Cell Death Protein 1 Inhibitors)) OR (PD-1 Inhibitor)) OR (Inhibitor, PD-1)) OR (PD 1 Inhibitor)) OR (Immune Checkpoint Blockade)) OR (Checkpoint Blockade, Immune)) OR (Immune Checkpoint Inhibition)) OR (Checkpoint Inhibition, Immune)) OR (PD-L1 Inhibitors)) OR (PD L1 Inhibitors)) OR (Programmed Death-Ligand 1 Inhibitors)) OR (Programmed Death Ligand 1 Inhibitors)) OR (PD-L1 Inhibitor)) OR (PD L1 Inhibitor)) OR (PD-1-PD-L1 Blockade)) OR (Blockade, PD-1-PD-L1)) OR (PD 1 PD L1 Blockade)) OR (Toripalimab)) OR (Sintilimab)) OR (Camrelizumab)) OR (Tislelizumab)) OR (Cindilimab)) OR (Penpulimab)) OR (Serplulimab)) OR (Pucotenlimab)) OR (Pembrolizumab)) OR (Nivolumab)) OR (Sugemalimab)) OR (Envafolimab)) OR (Adebrelimab)) OR (Atezolizumab)) OR (Durvalumab) AND (1000/1/1:2026/2/1[pdat]) | 89918 |
| 2 | ((((((((((((((((((((((((((((((((Angiogenesis Factor Inhibitor) OR (Angiogenesis Inhibitors)) OR (Angiogenesis Factor Inhibitors)) OR (Angiogenesis Inhibitor)) OR (Angiogenetic Antagonist)) OR (Angiogenetic Antagonists)) OR (Angiogenetic Inhibitor)) OR (Angiogenetic Inhibitors)) OR (Angiogenic Antagonist)) OR (Angiogenic Antagonists)) OR (Angiogenic Inhibitor)) OR (Angiogenic Inhibitors)) OR (Angiostatic Agent)) OR (Angiostatic Agents)) OR (Antagonists, Angiogenic)) OR (Anti-Angiogenesis Effect)) OR (Anti-Angiogenesis Effects)) OR (Anti-Angiogenetic Agent)) OR (Anti-Angiogenetic Agents)) OR (Anti-Angiogenic Drug)) OR (Anti-Angiogenic Drugs)) OR (Antiangiogenesis Effect)) OR (Antiangiogenesis Effects)) OR (Antiangiogenic Agent)) OR (Antiangiogenic Agents)) OR (Inhibitors, Angiogenesis)) OR (Inhibitors, Angiogenetic)) OR (Inhibitors, Angiogenic)) OR (Inhibitors, Neovascularization)) OR (Neovascularization Inhibitor)) OR (Neovascularization Inhibitors)) OR (Bevacizumab)) OR (Ramucirumab) AND (1000/1/1:2026/2/1[pdat]) | 137766 |
| 3 | (((((((((((Carcinoma, Non-Small-Cell Lung[MeSH Terms]) OR (Carcinoma, Non Small Cell Lung)) OR (Carcinomas, Non-Small-Cell Lung)) OR (Lung Carcinoma, Non-Small-Cell)) OR (Lung Carcinomas, Non-Small-Cell)) OR (Non-Small-Cell Lung Carcinomas)) OR (Carcinoma, Non-Small Cell Lung)) OR (Non-Small Cell Lung Cancer)) OR (Non-Small-Cell Lung Carcinoma)) OR (Non Small Cell Lung Carcinoma)) OR (Nonsmall Cell Lung Cancer)) OR (Non-Small Cell Lung Carcinoma) AND (1000/1/1:2026/2/1[pdat]) | 134167 |
| 4 | ((((((Ivonescimab) OR (PD-1/VEGF bispecific antibody)) OR (PD-L1/VEGF bispecific antibody)) OR (PD-1/VEGF dual antibody)) OR (PD-L1/VEGF dual antibody)) OR (bispecific PD-1/VEGF)) OR (bispecific PD-L1/VEGF) AND (1000/1/1:2026/2/1[pdat]) | 55 |
| 5 | #1 AND #2 OR #4 | 5780 |
| 6 | #5 AND #3 | 749 |

**Web of Science**

| Search number | Search Details | Results |
| --- | --- | --- |
| 1 | TS=(Angiogenesis Factor Inhibitor OR Angiogenesis Inhibitors OR Angiogenesis Factor Inhibitors OR Angiogenesis Inhibitor OR Angiogenetic Antagonist OR Angiogenetic Antagonists OR Angiogenetic Inhibitor OR Angiogenetic Inhibitors OR Angiogenic Antagonist OR Angiogenic Antagonists OR Angiogenic Inhibitor OR Angiogenic Inhibitors OR Angiostatic Agent OR Angiostatic Agents OR Antagonists, Angiogenic OR Anti-Angiogenesis Effect OR Anti-Angiogenesis Effects OR Anti-Angiogenetic Agent OR Anti-Angiogenetic Agents OR Anti-Angiogenic Drug OR Anti-Angiogenic Drugs OR Antiangiogenesis Effect OR Antiangiogenesis Effects OR Antiangiogenic Agent OR Antiangiogenic Agents OR Inhibitors, Angiogenesis OR Inhibitors, Angiogenetic OR Inhibitors, Angiogenic OR Inhibitors, Neovascularization OR Neovascularization Inhibitor OR Neovascularization Inhibitors OR Bevacizumab OR Ramucirumab) AND PY=(1000-2026) | 80432 |
| 2 | TS=(Immune Checkpoint Blockade OR Immune Checkpoint Inhibitors OR Immune Checkpoint Blockers OR Immune Checkpoint Inhibition OR Immune Checkpoint Inhibitor OR PD-1 Inhibitor OR PD-1 Inhibitors OR PD-1-PD-L1 Blockade OR PD-L1 Inhibitor OR PD-L1 Inhibitors OR Programmed Cell Death Protein 1 Inhibitor OR Programmed Cell Death Protein 1 Inhibitors OR Programmed Death-Ligand 1 Inhibitors OR Toripalimab OR Sintilimab OR Camrelizumab OR Tislelizumab OR Cindilimab OR Penpulimab OR Serplulimab OR Pucotenlimab OR Pembrolizumab OR Nivolumab OR Sugemalimab OR Envafolimab OR Adebrelimab OR Atezolizumab OR DurvalumabImmune Checkpoint Blockade OR Immune Checkpoint Inhibitors OR Immune Checkpoint Blockers OR Immune Checkpoint Inhibition OR Immune Checkpoint Inhibitor OR PD-1 Inhibitor OR PD-1 Inhibitors OR PD-1-PD-L1 Blockade OR PD-L1 Inhibitor OR PD-L1 Inhibitors OR Programmed Cell Death Protein 1 Inhibitor OR Programmed Cell Death Protein 1 Inhibitors OR Programmed Death-Ligand 1 Inhibitors OR Toripalimab OR Sintilimab OR Camrelizumab OR Tislelizumab OR Cindilimab OR Penpulimab OR Serplulimab OR Pucotenlimab OR Pembrolizumab OR Nivolumab OR Sugemalimab OR Envafolimab OR Adebrelimab OR Atezolizumab OR Durvalumab) AND PY=(2000-2026) | 112735 |
| 3 | TS=(Ivonescimab OR PD-1/VEGF bispecific antibody OR PD-L1/VEGF bispecific antibody OR PD-1/VEGF dual antibody OR PD-L1/VEGF dual antibody OR bispecific PD-1/VEGF OR bispecific PD-L1/VEGF) AND PY=(2000-2026) | 97 |
| 4 | TS=(Carcinoma, Non-Small-Cell Lung OR Carcinoma, Non Small Cell Lung OR Carcinomas, Non-Small-Cell Lung OR Lung Carcinoma, Non-Small-Cell OR Lung Carcinomas, Non-Small-Cell OR Non-Small-Cell Lung Carcinomas OR Carcinoma, Non-Small Cell Lung OR Non-Small Cell Lung Cancer OR Non-Small-Cell Lung Carcinoma OR Non Small Cell Lung Carcinoma OR Nonsmall Cell Lung Cancer OR Non-Small Cell Lung Carcinoma OR Lung Neoplasms OR Neoplasms, Pulmonary OR Neoplasm, Pulmonary OR Pulmonary Neoplasm OR Pulmonary Neoplasms OR Neoplasms, Lung OR Lung Neoplasm OR Neoplasm, Lung OR Lung Cancer OR Cancer, Lung OR Cancers, Lung OR Lung Cancers OR Cancer of Lung OR Pulmonary Cancer OR Cancer, Pulmonary OR Cancers, Pulmonary OR Pulmonary Cancers OR Cancer of the Lung) AND PY=(2000-2026) | 398759 |
| 5 | (#1 AND #2 OR #3) AND #4 | 1302 |

The search was limited to publications between 1 January 2000 and 1 February 2026 using WOS Custom date range.

**Embase**

| Search number | Search Details | Results |
| --- | --- | --- |
| 1 | ('immune checkpoint blocker'/exp OR 'immune checkpoint inhibitors':ab,ti OR 'immune checkpoint inhibitor':ab,ti OR 'toripalimab':ab,ti OR 'sintilimab':ab,ti OR 'camrelizumab':ab,ti OR 'tislelizumab':ab,ti OR 'cindilimab':ab,ti OR 'penpulimab':ab,ti OR 'serplulimab':ab,ti OR 'pucotenlimab':ab,ti OR 'pembrolizumab':ab,ti OR 'nivolumab':ab,ti OR 'sugemalimab':ab,ti OR 'envafolimab':ab,ti OR 'adebrelimab':ab,ti OR 'atezolizumab':ab,ti OR 'durvalumab':ab,ti) AND [<1966-2026]/py | 138994 |
| 2 | ('angiogenesis inhibitors'/exp OR 'angiostatic agent':ab,ti OR 'angiostatic drug':ab,ti OR 'anti angiogenesis agent':ab,ti OR 'anti angiogenesis drug':ab,ti OR 'anti angiogenic agent':ab,ti OR 'anti angiogenic drug':ab,ti OR 'antiangiogenesis agent':ab,ti OR 'antiangiogenesis drug':ab,ti OR 'antiangiogenic agent':ab,ti OR 'antiangiogenic drug':ab,ti OR 'neovascularisation inhibitor':ab,ti OR 'neovascularization inhibitor':ab,ti OR 'tumor vascularization inhibitor':ab,ti OR 'tumour vascularisation inhibitor':ab,ti OR 'vascularisation inhibitor':ab,ti OR 'vascularization inhibitor':ab,ti OR 'angiogenesis inhibitor':ab,ti OR 'bevacizumab':ab,ti OR 'ramucirumab':ab,ti) AND [<1966-2026]/py | 681627 |
| 3 | ('non small cell lung cancer'/exp OR 'bronchial non small cell cancer':ab,ti OR 'bronchial non small cell carcinoma':ab,ti OR 'carcinoma, non-small-cell lung':ab,ti OR 'lung cancer, non small cell':ab,ti OR 'lung non small cell cancer':ab,ti OR 'lung non small cell carcinoma':ab,ti OR 'non oat cell lung cancer':ab,ti OR 'non small cell bronchial cancer':ab,ti OR 'non small cell cancer, lung':ab,ti OR 'non small cell lung carcinoma':ab,ti OR 'non small cell pulmonary cancer':ab,ti OR 'non small cell pulmonary carcinoma':ab,ti OR 'non squamous nsclc':ab,ti OR 'non-oat cell lung cancer':ab,ti OR 'non-small-cell lung carcinoma':ab,ti OR 'nonsmall cell carcinoma of the lung':ab,ti OR 'nonsmall cell lung cancer':ab,ti OR 'nonsmall cell lung carcinoma':ab,ti OR 'pulmonary non small cell cancer':ab,ti OR 'pulmonary non small cell carcinoma':ab,ti OR 'non small cell lung cancer':ab,ti) AND [<1966-2026]/py | 291244 |
| 4 | ('ivonescimab':ab,ti OR 'pd-1/vegf bispecific antibody':ab,ti OR 'pd-l1/vegf bispecific antibody':ab,ti OR 'pd-1/vegf dual antibody':ab,ti OR 'pd-l1/vegf dual antibody':ab,ti OR 'bispecific pd-1/vegf':ab,ti OR 'bispecific pd-l1/vegf':ab,ti) AND [<1966-2026]/py | 253 |
| 5 | #1 AND #2 | 35372 |
| 6 | #4 OR #5 | 35555 |
| 7 | #3 AND #6 | 7492 |

The literature search in Embase was conducted up to 1 February 2026. After excluding publications beyond this date, a total of 7031 records were retained for analysis.

**Cochrane**

| Search number | Search Details | Results |
| --- | --- | --- |
| 1 | MeSH descriptor: [Immune Checkpoint Inhibitors] explode all trees | 487 |
| 2 | (immune checkpoint inhibitors OR immune checkpoint blockers OR programmed death-ligand 1 inhibitors OR PD-L1 inhibitors OR nivolumab OR pembrolizumab OR dostarlimab OR durvalumab OR atezolizumab OR avelumab OR treprizumab OR sintilimab OR camrelizumab OR tremelimumab OR zimberelimab OR penpulimab OR serplulimab OR pucotenlimab OR envafolimab OR adebrelimab OR ipilimumab OR PD-1 OR Programmed cell death-ligand 1 OR Programmed cell death-1 OR PD-L1 OR Toripalimab OR Tislelizumab OR Cemiplimab OR Sugemalimab):ti,ab,kw | 16966 |
| 3 | #1 OR #2 | 16966 |
| 4 | MeSH descriptor: [Angiogenesis Inhibitors] explode all trees | 2112 |
| 5 | (Angiogenesis Factor Inhibitor OR Angiogenesis Inhibitors OR Angiogenesis Factor Inhibitors OR Angiogenesis Inhibitor OR Angiogenetic Antagonist OR Angiogenetic Antagonists OR Angiogenetic Inhibitor OR Angiogenetic Inhibitors OR Angiogenic Antagonist OR Angiogenic Antagonists OR Angiogenic Inhibitor OR Angiogenic Inhibitors OR Angiostatic Agent OR Angiostatic Agents OR Antagonists, Angiogenic OR Anti-Angiogenesis Effect OR Anti-Angiogenesis Effects OR Anti-Angiogenetic Agent OR Anti-Angiogenetic Agents OR Anti-Angiogenic Drug OR Anti-Angiogenic Drugs OR Antiangiogenesis Effect OR Antiangiogenesis Effects OR Antiangiogenic Agent OR Antiangiogenic Agents OR Inhibitors, Angiogenesis OR Inhibitors, Angiogenetic OR Inhibitors, Angiogenic OR Inhibitors, Neovascularization OR Neovascularization Inhibitor OR Neovascularization Inhibitors Bevacizumab OR Ramucirumab OR Vascular Endothelial Growth Factor A OR VEGF OR Vascular Endothelial Growth Factor OR Vascular Permeability Factor OR Vasculotropin OR VEGFR OR Bevacizumab OR Ramucirumab):ti,ab,kw | 18294 |
| 6 | #4 OR #5 | 18294 |
| 7 | (Ivonescimab OR PD-1/VEGF bispecific antibody OR PD-L1/VEGF bispecific antibody OR PD-1/VEGF dual antibody OR PD-L1/VEGF dual antibody OR bispecific PD-1/VEGF OR bispecific PD-L1/VEGF):ti,ab,kw | 63 |
| 8 | MeSH descriptor: [Carcinoma, Non-Small-Cell Lung] explode all trees | 7065 |
| 9 | (‘Non-Small-Cell Lung Carcinoma’ OR ‘Lung Carcinomas, Non-Small-Cell’ OR ‘Carcinoma, Non Small Cell Lung’ OR ‘Carcinoma, Non-Small Cell Lung’ OR ‘Non-Small-Cell Lung Carcinomas’ OR ‘Lung Carcinoma, Non-Small-Cell’ OR ‘Non Small Cell Lung Carcinoma’ OR ‘Non-Small Cell Lung Cancer’ OR ‘Carcinomas, Non-Small-Cell Lung’ OR ‘Nonsmall Cell Lung Cancer’ OR ‘Non-Small Cell Lung Carcinoma’):ti,ab,kw | 18944 |
| 10 | #8 OR #9 | 18944 |
| 11 | #3 AND #6 | 1834 |
| 12 | #11 OR #7 | 1861 |
| 13 | #12 AND #10 | 332 |

The literature search in Cochrane Library was conducted up to 1 February 2026. After excluding publications beyond this date, a total of 304 records were retained for analysis.

**ClinicalTrials.gov**

| Search number | Search Details | Results |
| --- | --- | --- |
| 1 | Condition/disease: "Non-Small Cell Lung Cancer NSCLC" OR "Non-small-cell Lung Cancer \(NSCLC\) Stage IV" OR "Non-small Cell Lung Cancer Stage IV" OR "Non-Small-Cell Lung Carcinoma" OR "Non Small Cell Lung Carcinoma NSCLC" OR "Non-small Cell Lung Cancer Metastatic" OR "Non-small Cell Lung Cancer Stage IIIB" OR "Non-small Cell Lung Cancer Stage IIIC" OR "Non-Small Cell Lung Cancer" | / |
| 2 | Intervention/treatment: (Immune Checkpoint Blockade OR Immune Checkpoint Inhibitors OR Immune Checkpoint Blockers OR Immune Checkpoint Inhibition OR Immune Checkpoint Inhibitor OR PD-1 Inhibitor OR PD-1 Inhibitors OR PD-1-PD-L1 Blockade OR PD-L1 Inhibitor OR PD-L1 Inhibitors OR Programmed Cell Death Protein 1 Inhibitor OR Programmed Cell Death Protein 1 Inhibitors OR Programmed Death-Ligand 1 Inhibitors OR Toripalimab OR Sintilimab OR Camrelizumab OR Tislelizumab OR Cindilimab OR Penpulimab OR Serplulimab OR Pucotenlimab OR Pembrolizumab OR Nivolumab OR Sugemalimab OR Envafolimab OR Adebrelimab OR Atezolizumab OR DurvalumabImmune Checkpoint Blockade OR Immune Checkpoint Inhibitors OR Immune Checkpoint Blockers OR Immune Checkpoint Inhibition OR Immune Checkpoint Inhibitor OR PD-1 Inhibitor OR PD-1 Inhibitors OR PD-1-PD-L1 Blockade OR PD-L1 Inhibitor OR PD-L1 Inhibitors OR Programmed Cell Death Protein 1 Inhibitor OR Programmed Cell Death Protein 1 Inhibitors OR Programmed Death-Ligand 1 Inhibitors OR Toripalimab OR Sintilimab OR Camrelizumab OR Tislelizumab OR Cindilimab OR Penpulimab OR Serplulimab OR Pucotenlimab OR Pembrolizumab OR Nivolumab OR Sugemalimab OR Envafolimab OR Adebrelimab OR Atezolizumab OR Durvalumab) AND (Angiogenesis Factor Inhibitor OR Angiogenesis Inhibitors OR Angiogenesis Factor Inhibitors OR Angiogenesis Inhibitor OR Angiogenetic Antagonist OR Angiogenetic Antagonists OR Angiogenetic Inhibitor OR Angiogenetic Inhibitors OR Angiogenic Antagonist OR Angiogenic Antagonists OR Angiogenic Inhibitor OR Angiogenic Inhibitors OR Angiostatic Agent OR Angiostatic Agents OR Antagonists, Angiogenic OR Anti-Angiogenesis Effect OR Anti-Angiogenesis Effects OR Anti-Angiogenetic Agent OR Anti-Angiogenetic Agents OR Anti-Angiogenic Drug OR Anti-Angiogenic Drugs OR Antiangiogenesis Effect OR Antiangiogenesis Effects OR Antiangiogenic Agent OR Antiangiogenic Agents OR Inhibitors, Angiogenesis OR Inhibitors, Angiogenetic OR Inhibitors, Angiogenic OR Inhibitors, Neovascularization OR Neovascularization Inhibitor OR Neovascularization Inhibitors OR Bevacizumab OR Ramucirumab) OR (Ivonescimab OR PD-1/VEGF bispecific antibody OR PD-L1/VEGF bispecific antibody OR PD-1/VEGF dual antibody OR PD-L1/VEGF dual antibody OR bispecific PD-1/VEGF OR bispecific PD-L1/VEGF) | / |
| 3 | "Non-Small Cell Lung Cancer NSCLC" OR "Non-small-cell Lung Cancer \(NSCLC\) Stage IV" OR "Non-small Cell Lung Cancer Stage IV" OR "Non-Small Cell Lung Carcinoma" OR "Non Small Cell Lung Carcinoma NSCLC" OR "Non-small Cell Lung Cancer Metastatic" OR "Non-small Cell Lung Cancer Stage IIIB" OR "Non-small Cell Lung Cancer Stage IIIC" OR "Non-Small Cell Lung Cancer" \| (Immune Checkpoint Blockade OR Immune Checkpoint Inhibitors OR Immune Checkpoint Blockers OR Immune Checkpoint Inhibition OR Immune Checkpoint Inhibitor OR PD-1 Inhibitor OR PD-1 Inhibitors OR PD-1-PD-L1 Blockade OR PD-L1 Inhibitor OR PD-L1 Inhibitors OR Programmed Cell Death Protein 1 Inhibitor OR Programmed Cell Death Protein 1 Inhibitors OR Programmed Death-Ligand 1 Inhibitors OR Toripalimab OR Sintilimab OR Camrelizumab OR Tislelizumab OR Cindilimab OR Penpulimab OR Serplulimab OR Pucotenlimab OR Pembrolizumab OR Nivolumab OR Sugemalimab OR Envafolimab OR Adefrelimab OR Atezolizumab OR DurvalumabImmune Checkpoint Blockade OR Immune Checkpoint Inhibitors OR Immune Checkpoint Blockers OR Immune Checkpoint Inhibition OR Immune Checkpoint Inhibitor OR PD-1 Inhibitor OR PD-1 Inhibitors OR PD-1-PD-L1 Blockade OR PD-L1 Inhibitor OR PD-L1 Inhibitors OR Programmed Cell Death Protein 1 Inhibitor OR Programmed Cell Death Protein 1 Inhibitors OR Programmed Death-Ligand 1 Inhibitors OR Toripalimab OR Sintilimab OR Camrelizumab OR Tislelizumab OR Cindilimab OR Penpulimab OR Serplulimab OR Pucotenlimab OR Pembrolizumab OR Nivolumab OR Sugemalimab OR Envafolimab OR Adefrelimab OR Atezolizumab OR Durvalumab) AND (Angiogenesis Factor Inhibitor OR Angiogenesis Inhibitors OR Angiogenesis Factor Inhibitors OR Angiogenesis Inhibitor OR Angiogenetic Antagonist OR Angiogenetic Antagonists OR Angiogenetic Inhibitor OR Angiogenetic Inhibitors OR Angiogenic Antagonist OR Angiogenic Antagonists OR Angiogenic Inhibitor OR Angiogenic Inhibitors OR Angiostatic Agent OR Angiostatic Agents OR Antagonists, Angiogenic OR Anti-Angiogenesis Effect OR Anti-Angiogenesis Effects OR Anti-Angiogenetic Agent OR Anti-Angiogenetic Agents OR Anti-Angiogenic Drug OR Anti-Angiogenic Drugs OR Antiangiogenesis Effect OR Antiangiogenesis Effects OR Antiangiogenic Agent OR Antiangiogenic Agents OR Inhibitors, Angiogenesis OR Inhibitors, Angiogenetic OR Inhibitors, Angiogenic OR Inhibitors, Neovascularization OR Neovascularization Inhibitor OR Neovascularization Inhibitors OR Bevacizumab OR Ramucirumab) OR (Ivonescimab OR PD-1/VEGF bispecific antibody OR PD-L1/VEGF bispecific antibody OR PD-1/VEGF dual antibody OR PD-L1/VEGF dual antibody OR bispecific PD-1/VEGF OR bispecific PD-L1/VEGF) \| Study start on or before 02/01/2026 | 142 |
